# Controllable Generation of Pathogen‐Specific Antimicrobial Peptides Through Knowledge‐Aware Prompt Diffusion Model

**DOI:** 10.1002/advs.202507457

**Published:** 2025-09-15

**Authors:** Yongkang Wang, Menglu Li, Feng Huang, Minyao Qiu, Wen Zhang

**Affiliations:** ^1^ College of Informatics Huazhong Agricultural University No.1, Shizishan Street Wuhan 430070 China

**Keywords:** controllable generation, deep learning, pathogen‐specific, peptide discovery, prompt diffusion

## Abstract

Generative models have shown considerable promise in antimicrobial peptide design; however, their ability to generate pathogen‐specific peptides remains limited due to data scarcity. In this study, KPPepGen, a controllable generative framework that leverages knowledge‐aware pathogen prompts derived from pre‐training on Gene Ontology and pathogen knowledge graphs, which act as knowledge injections to guide a diffusion model in generating biologically plausible peptides tailored to specific pathogens, is introduced. Then, KPPepGen is extended for peptide optimization by integrating prompt‐guided partial diffusion with multi‐site combinatorial mutations. Experimental results show that KPPepGen can simultaneously generate valid peptides for 56 distinct pathogens, achieving high novelty, favorable physicochemical properties, and delivering over a 10% performance improvement for pathogens with limited training data. Further analysis demonstrates that KPPepGen effectively captures essential sequence and structure patterns characteristic of individual pathogens. The optimization results reveal a high success rate of 44.3% for Magainin 2, along with an average improvement of 7.6% compared to the ESM‐based method, underscoring the effectiveness of KPPepGen in enhancing the overall performance of peptides. Finally, for clinically relevant pathogens such as *E. coli* and *S. aureus*, KPPepGen successfully generated nine novel peptides that exhibit strong antimicrobial activity and low cytotoxicity in the wet‐lab evaluation.

## Graphical abstract

KPPepGen is a knowledge‐aware prompt diffusion model for the controllable generation of pathogen‐specific AMPs. It outperforms existing methods and is capable of simultaneously generating peptides for distinct pathogens, demonstrating superior performance with favorable properties and docking efficacy. Wet‐lab evaluations confirm that novel AMPs generated by KPPepGen are effective for given pathogen requirements, exhibiting potent antimicrobial efficacy alongside favorable cytotoxicity property.

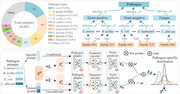

## 1 Introduction

While the emergence of antibiotics has been crucial in the treatment of bacterial infections, their misuse has led to a growing problem of bacterial resistance.^[^ ^1^ ^]^ As bacterial resistance evolves faster than new antibiotics can be developed, human health faces a serious threat, pushing us toward a post‐antibiotic era.^[^ ^2^ ^]^ Antimicrobial peptides (AMPs), short sequences of 5 to 50 amino acids, offer effective antimicrobial activity and low cytotoxicity, making them a promising alternative, especially against antibiotic‐resistant infections.^[^ ^3^, ^4^, ^5^ ^]^ Consequently, AMPs have become a global research focus with the potential to be next‐generation antibacterial drugs. However, designing novel and function‐specific AMPs remains a considerable challenge.^[^ ^6^ ^]^

Traditional approaches for discovering AMPs, such as phage display technologies, involve conducting a comprehensive screening of peptides,^[^ ^7^, ^8^ ^]^ but they are usually costly and time‐intensive. In recent years, with the accumulation of AMP data, artificial intelligence (AI) technologies, especially generative models, have gained significant attention for exploring the sequence space to design novel AMPs.^[^ ^9^, ^10^, ^11^ ^]^ These peptide generative models can be broadly classified into four distinct approaches.^[^ ^12^ ^]^ Specifically, the positive‐only learning approaches predominantly capture the underlying distribution of given training data to generate new peptides similar to those in the training set. For example, AMPGen^[^ ^13^ ^]^ generates novel peptide sequences using an encoder‐decoder architecture trained on existing AMP datasets. The discriminator‐guided filtering approaches produce peptides that require subsequent screening based on given criteria (e.g., physicochemical properties and antimicrobial activity) to eliminate inactive sequences,^[^ ^14^, ^15^ ^]^ leading to inefficiencies in the design process. For instance, AMPGAN^[^ ^16^ ^]^ employs a generative adversarial network to produce novel peptide sequences, which are subsequently re‐evaluated using property evaluation tools, such as modlAMP,^[^ ^17^ ^]^ ensuring the generated sequences with desirable characteristics. Meanwhile, the latent space sampling approaches (e.g., WAE‐PSO^[^ ^18^ ^]^) and conditional generation with AMP generator (e.g., HydrAMP^[^ ^19^ ^]^) integrate additional constraint information, such as property labels and antimicrobial discriminator, allowing them to guide the peptide distribution relevant to given constraints. Despite promising advances, a critical challenge persists: pathogens exhibit considerable diversity, necessitating the development of antimicrobial methods that are effective across a wide range of specific pathogens.^[^ ^20^ ^]^ The common generation strategy (i.e., one‐against‐one) relies on single‐task training for individual pathogens, which is inherently inefficient and lacks transferability, requiring model retraining whenever a new pathogen is introduced. Additionally, several approaches involve training a peptide generator using known peptide data (e.g., AMPs and non‐AMPs) to produce new peptides, followed by the property evaluation tools to filter out inactive sequences for specific pathogens, thereby incurring substantial computational and temporal costs. Notably, while genomic databases have cataloged ≈1000 pathogens,^[^ ^21^ ^]^ AMPs have been experimentally validated for fewer than 8% of these (≈80 pathogens). Most of these pathogens have limited known AMPs (just 17 pathogens with over 500 AMPs), revealing a stark disparity in AMP data availability across pathogens, which significantly restricts the applicability of existing peptide generation methods, confining their effectiveness to a narrow range of pathogens. Therefore, our study aims to develop a generative model that can efficiently generate peptides for various pathogen‐specific tasks simultaneously (i.e., one‐against‐all), thereby eliminating the need for repeated training and re‐evaluation.

Indeed, AMPs possess extensive domain‐specific knowledge, involving biological correlations among AMPs and functional divergences across different pathogens,^[^ ^22^, ^23^ ^]^ which could elucidate pathogen biases relevant to AMPs.^[^ ^24^ ^]^ Additionally, the general knowledge embeddings^[^ ^25^, ^26^ ^]^ derived from Gene Ontology (GO) encompass millions of structured biological facts, while the knowledge graph provides hierarchical tree structures to link standard GO classes and bio‐entities.^[^ ^27^ ^]^ For instance, the Cut&CLIP framework employs a contrastive learning strategy to align the peptide knowledge with their corresponding sequences within a feature space, which facilitates the integration of complex biological information, thereby enhancing the model's downstream generative capabilities.^[^ ^28^ ^]^ Notably, incorporating such peptide knowledge not only improves the quality of peptide representation but also serves as a robust prompt for various biological applications. In recent years, we have witnessed the successful applications of prompt learning in the protein design,^[^ ^14^, ^29^ ^]^ which craft effective prompts that maximize the ability of models to understand contextual nuances of a given task, guiding them toward generating desired outputs.^[^ ^10^, ^30^ ^]^ By integrating pathogen‐specific knowledge embeddings as prompts, it becomes possible to develop generative models with improved transferability across pathogens, achieving the controllable generation tailored to specific pathogens.

In this study, we introduce KPPepGen, a novel knowledge‐aware prompt diffusion model for the controllable generation of pathogen‐specific antimicrobial peptides. Leveraging peptide data from the UniProt database and AMP datasets, we constructed the GO and pathogen knowledge graphs and conducted the knowledge‐aware pre‐training process to learn pathogen embeddings, which served as prompts for each corresponding pathogen. Then, KPPepGen employed the learned pathogen prompts and integrated the prompt‐guided strategy with classifier‐free guidance diffusion to generate biologically plausible peptides tailored to specific pathogens. Experimental results demonstrate that KPPepGen significantly outperforms baselines, producing AMPs with superior properties and docking efficacy, particularly excelling at pathogen‐specific tasks. Further analysis shows that KPPepGen effectively identifies essential sequence and structure patterns unique to individual pathogens. Additionally, we extended the applicability of KPPepGen to peptide optimization by incorporating prompt‐guided partial diffusion, demonstrating its potential to enhance the properties of both natural and human AMPs. The wet‐lab evaluation further confirms the strong antimicrobial activity and low cytotoxicity of nine novel peptides generated by KPPepGen. Overall, KPPepGen represents a significant advancement in pathogen‐specific AMP generation.

## 2 Results

### 2.1 Overall Framework

**Figure 1:**
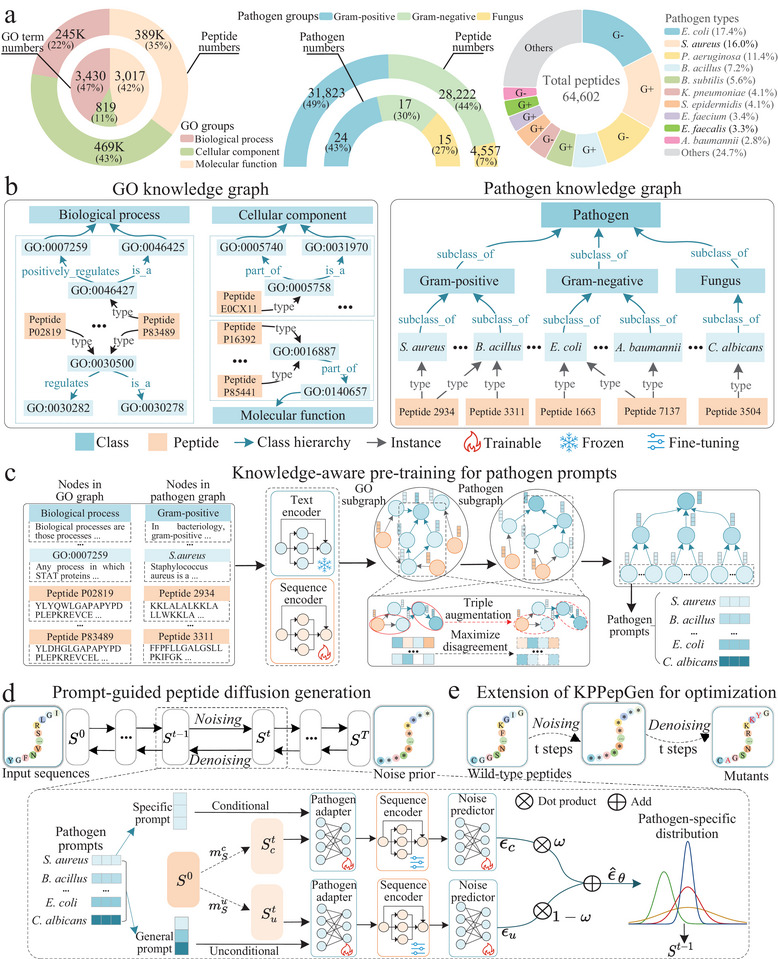
Overall of the knowledge‐aware prompt diffusion framework. a) Landscape of peptide data within GO and pathogen knowledge. b) A snapshot of GO/pathogen knowledge graphs. These graphs comprise class and peptide nodes, along with biological relationships among them. Class nodes are represented by textual descriptions of GO and pathogen terms, while peptide nodes correspond to amino acid sequences. c) Nodes in GO/pathogen graphs are encoded using two distinct modules: a frozen text encoder for class entities and a trainable sequence encoder for peptide entities. The GO/pathogen subgraphs are pre‐trained to maximize the disagreement between positive/negative triple instances, thereby learning embeddings of entities. Pathogen embeddings are extracted from the pathogen knowledge graph to serve as prompts. d) Prompt‐guided peptide diffusion consists of conditional and unconditional components, utilizing the pathogen prompt and pathogen marginal noise. e) Partial diffusion‐based optimization framework. The process begins by noising wild‐type peptides through forward diffusion, followed by denoising via backward diffusion to mutants.

The proposed KPPepGen framework is illustrated in **Figure** **1**. First, we curated the peptide dataset alongside GO and pathogen annotations, clearly depicting the data landscape (Figure 1a). Next, GO and pathogen knowledge graphs were constructed, containing triples that connect class and peptide entities (Figure 1b). The representations of nodes in these graphs were encoded using a text encoder and a sequence encoder. We then implemented a knowledge‐aware pre‐training to enhance the quality of class and peptide representations, while also learning pathogen prompts (Figure 1c). In the prompt‐guided peptide diffusion framework (Figure 1d), peptide sequences were generated under the guidance of learned pathogen‐specific prompts and predefined pathogen marginal distributions integrated within the diffusion process. The diffusion generation comprises both unconditional and conditional steps, utilizing a module consisting of a pathogen adapter, a pre‐trained peptide sequence encoder, and a noise predictor to effectively map inputs to pathogen‐specific distributions. Then, KPPepGen is extended for peptide optimization by integrating prompt‐guided partial diffusion (Figure 1e).

### 2.2 KPPepGen Outperforms the State‐of‐the‐Art Methods in Pathogen‐Specific Peptide Generation

In this section, we compared our KPPepGen with a variety of state‐of‐the‐art (SOAT) methods. Each method was used to generate 2000 peptides for each of the 56 specific pathogens. The resulting peptides were evaluated using a comprehensive set of property metrics, including sequence similarity, instability, transmembrane tendency (TM_tend), and four physicochemical properties. Additionally, their binding scores were quantified through molecular docking against both general and specific targets for the pathogens.

**Figure 2:**
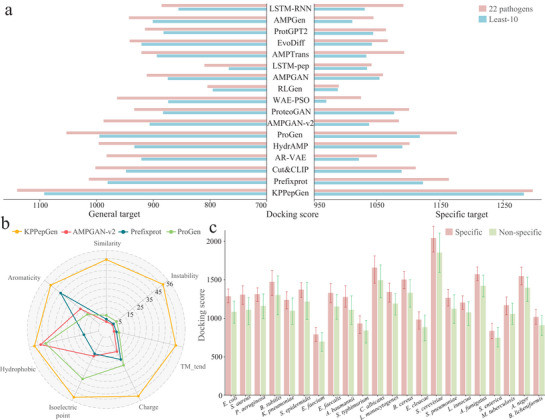
Performance comparison of KPPepGen and baseline methods. a) Docking scores of KPPepGen and baseline methods for general and pathogen‐specific targets. b) Number of individual pathogen tasks in which SOAT methods demonstrate best performance across seven metrics. c) Comparison of docking scores for pathogen‐specific and non‐specific peptide interactions from KPPepGen.

First, we compared the properties of peptides generated by different methods. As shown in **Table** **1**, conditional generation methods outperform other three categories of methods based on the average similarity, instability, and TM_tend scores across all 56 pathogens. Notably, KPPepGen facilitates simultaneous peptide generation for all 56 pathogens and achieves the lowest average similarity, instability, and TM_tend scores compared to other conditional generation methods employing the one‐against‐one strategy, outperforming the second‐best method (i.e., ProGen) by 14.8%, 6.7%, and 16.0% in terms of three metrics, respectively. Furthermore, we focused on the performance of KPPepGen in 10 pathogens with the lowest sample counts (least‐10), where pathogens constitute only 1.9% of total peptide dataset and 17.9% of the total pathogen count. While the performance of all methods declines on these pathogens compared to their overall performance across all 56 pathogens, KPPepGen demonstrates even greater relative advantages. Specifically, it improves upon the second‐best method by 15.6%, 7.4%, and 17.5% across similarity, instability, and TM_tend scores, underscoring its robust performance against pathogens with limited known peptides. The physicochemical properties (e.g., charge, isoelectric point, hydrophobic, and aromaticity) of natural AMPs typically fall within specific biological ranges, and significant deviations among generated peptides may compromise their biological plausibility. For example, although AMPs generally exhibit slightly higher charges compared to non‐AMPs, excessive charges can increase cytotoxicity, ultimately reducing antimicrobial efficacy.^[^ ^31^ ^]^ To investigate this, we conducted the statistical analysis comparing the distributions of each physicochemical property between generated and training peptides. As shown in Table 1, baselines frequently produce peptides whose physicochemical properties significantly deviate from natural AMPs, particularly among least‐10 pathogens. The second‐best method (i.e., ProGen) achieves alignment in only three of eight comparisons, whereas peptides generated by KPPepGen consistently align with training peptide profiles across all eight comparisons, highlighting the superior biological plausibility and robustness of our method. Notably, the second‐ and third‐best methods (i.e., ProGen and Prefixprot) within the conditional generation subgroup are originally protein‐based methods, whereas the remaining methods (e.g., HydrAMP and Cut&CLIP) are designed for peptide generation. This observation suggests that leveraging the protein sequence space can enhance the performance of peptide generation, while our KPPepGen achieves a comparable benefit by incorporating knowledge pretraining. Beyond evaluating overall performance, we assessed the method superiority at the individual pathogen. As shown in **Figure** **2**b, KPPepGen achieved the best performance in at least 49 out of 56 pathogen‐specific tasks, clearly outperforming baselines, underscoring the method's robustness and broad applicability across diverse pathogens.

**Table 1:** Performance comparison of KPPepGen and baseline methods for generated peptides.

Methods		Similarity ↓		Instability ↓		TM_tend ↓		Physicochemical property							
								Charge		Isoelectric		Hydrophobic		Aromaticity	
		All	Least‐10	All	Least‐10	All	Least‐10	All	Least‐10	All	Least‐10	All	Least‐10	All	Least‐10
Positive‐only learning	LSTM‐RNN	48.96	57.33	51.03	53.77	0.694	0.711	***	***	ns	*	**	***	*	*
	AMPGen	46.23	55.76	49.81	51.07	0.725	0.741	***	***	**	***	***	***	**	*
	${\text{ProtGPT2}}^{\#}$	46.45	54.12	49.11	50.62	0.733	0.749	***	***	***	***	***	***	***	***
	${\text{EvoDiff}}^{\#}$	46.23	55.66	48.37	49.42	0.588	0.612	*	**	*	**	***	***	**	***
Discriminator ‐guided filtering	AMPTrans	47.61	54.27	49.25	50.11	0.727	0.764	***	***	***	***	*	***	***	***
	LSTM‐Pep	48.15	56.53	49.97	52.62	0.719	0.757	***	***	***	***	***	***	*	**
	AMPGAN	51.19	59.70	51.72	54.04	0.740	0.788	**	**	***	***	***	***	***	***
	RLGen	46.72	54.39	50.13	52.88	0.746	0.750	***	***	***	***	*	**	ns	*
Latent space sampling	WAE‐PSO	56.91	63.28	49.34	50.75	0.753	0.766	***	***	***	***	***	***	**	**
	${\text{ProteoGAN}}^{\#}$	41.29	46.18	44.90	46.73	0.553	0.637	***	***	***	***	**	**	***	***
	AMPGAN‐v2	43.03	45.62	44.53	46.79	0.549	0.651	***	***	*	**	ns	ns	***	***
Conditional generation	${\text{ProGen}}^{†,\#}$	40.56	44.16	43.49	45.94	0.524	0.622	*	*	ns	**	ns	ns	*	**
	HydrAMP	41.95	45.07	44.02	46.47	0.572	0.667	***	***	***	***	***	**	**	***
	${\text{AR-VAE}}^{†,\#}$	41.93	47.85	45.45	47.05	0.554	0.658	**	*	***	***	**	**	***	***
	$\text{Cut}\&{\text{CLIP}}^{†}$	41.52	45.19	43.92	46.40	0.550	0.651	**	**	**	***	*	**	**	***
	${\text{Prefixprot}}^{†,\#}$	40.67	44.25	43.78	46.14	0.540	0.644	***	***	***	***	**	**	ns	ns
	KPPepGen†	**34.54**	**37.27**	**40.55**	**42.52**	**0.440**	**0.513**	ns	ns	ns	ns	ns	ns	ns	ns

The best‐performing results are marked in **bold** and the suboptimal results are underlined. The superscript ‘†’ indicates methods that support the one‐against‐all strategy in peptide generation, while all unlabeled methods follow the one‐against‐one strategy. ‘#’ denotes methods that were originally designed for protein generation and subsequently adapted for peptide design. ‘All’ indicates the average performance across all 56 pathogens. ns, no statistical significance; * p<5e‐2; ** p<1e‐2; *** p<1e‐3.

Antimicrobial peptides exert their inhibitory effects by binding to specific target proteins of pathogens, thereby disrupting their function.^[^ ^32^, ^33^ ^]^ Previous studies have demonstrated that screening peptides based on their binding affinity to pathogen target proteins is a viable strategy for AMP discovery.^[^ ^34^ ^]^ Herein, we employed trRosetta^[^ ^35^ ^]^ to predict the structures of generated peptides and utilized the molecular docking tool ZDOCK to evaluate their docking scores against pathogen targets. Given the limited availability of experimentally determined target structures in the PDB database, we focused our analysis on 22 pathogens with documented targets, including both general and specific targets (Table S1, Supporting Information). As illustrated in Figure 2a, peptides generated by KPPepGen consistently achieved higher average docking scores across these 22 pathogens compared to all baseline models, surpassing the second‐best method (ProGen) by 8.3% for general targets and 10.4% for specific targets. Among the 10 least‐sampled pathogens, KPPepGen showed even greater advantages, outperforming ProGen by 10.2% for general targets and 14.8% for specific targets. Furthermore, KPPepGen obtained the highest docking scores for 19 out of 22 pathogens with general targets and 18 out of 22 pathogens with specific targets, highlighting its robust performance across diverse pathogen‐specific contexts. Additionally, we analyzed the pathogen specificity of our generated peptides, by considering docking scores of 2000 generated peptides for each pathogen (pathogen‐specific) and randomly sampled 2000 generated peptides for the other 21 pathogens (non‐specific) with the pathogen‐specific target. As shown in Figure 2c, pathogen‐specific peptides consistently exhibit higher docking scores than non‐specific peptides, achieving an average of 12.8±2.2% improvement across these 22 pathogens. Remarkably, KPPepGen maintains a significant score advantage exceeding 11% even for least‐10 pathogens, demonstrating its ability to preserve high specificity across various pathogens.

Overall, these findings highlight the superior performance of KPPepGen in generating peptides with favorable properties, docking efficacy, and pathogen specificity across diverse pathogens. More results of ablation experiments can be found in Section S3.5 (Supporting Information).

### 2.3 KPPepGen Leverages Pathogen Prompts to Guide the Generation of Pathogen‐Specific Peptides

In this section, we comprehensively discussed how the knowledge‐aware pathogen prompts effectively guide pathogen‐specific peptide generation.

**Figure 3:**
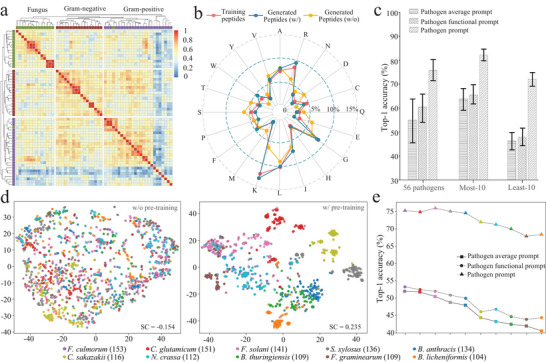
Statistical visualization for pathogen prompts and peptides. a) The heatmap depicts hierarchical clustering relationships among all 56 pathogen prompts. b) A radar chart contrasts amino acid frequency distributions between training peptides (red) and generated peptides with (blue) and without (yellow) prompt guidance. c) Top‐1 accuracy of three strategies for guiding peptide embeddings. d) The scatter plot with t‐SNE analysis to visualize peptide feature distributions for least‐10 pathogens without/with pre‐training assessed using the silhouette coefficient (SC). Parenthetical values denote peptide counts of given pathogens. e) Detailed results of top‐1 accuracy for least‐10 pathogens.

First, we employed the heatmap coupled with the hierarchical clustering analysis to examine relationships among 56 pathogen prompts learned through the knowledge‐aware pre‐training. As illustrated in **Figure** **3**a, these pathogens were effectively clustered into three distinct categories, including Gram‐positive, Gram‐negative, and Fungus, which align closely with their established microbial classifications, except for one fungus‐related prompt mistakenly grouped within the Gram‐positive category. Additionally, pathogen prompts exhibit notable pathogen‐specific distinctions, as indicated by the prominent diagonal line (highlighted in red) in Figure 3a. Meanwhile, these prompts also display strong intra‐group consistency and clear inter‐group differentiation. This embedding‐space differentiation underscores how knowledge‐aware pre‐training facilitates the semantic representation of pathogens, enabling the model to effectively capture both shared characteristics and specific differences across diverse pathogens.

Subsequently, we assessed the overall compositional consistency between the training data and the generated peptides through comparative frequency analysis of their amino acid profiles. As illustrated in Figure 3b, peptides generated with prompt guidance show amino acid frequency distributions closely aligned with the training peptides, reflected by a low overall frequency error of just 12%. This alignment is particularly pronounced for arginine (R) and lysine (K), consistent with their selective enrichment as cationic residues commonly found in AMPs (Figure S1a, Supporting Information). Conversely, peptides generated without prompt guidance exhibit substantially different amino acid distributions, resulting in a significantly higher overall frequency error of 32%. This substantial discrepancy underscores the effectiveness of prompt guidance in maintaining biologically relevant compositional characteristics.

Furthermore, we investigated whether pathogen prompts could effectively classify peptides into their corresponding pathogens by calculating the cosine similarity between peptide embeddings and pathogen prompts, assigning each peptide to the pathogen with the highest similarity. For comparison, we evaluated two alternative prompt representations: 1) pathogen average prompts, defined as the mean embedding of training peptides associated with each pathogen; and 2) pathogen functional prompts, derived from the textual description of each pathogen and encoded using PubMedBERT. As shown in Figure 3c, across all 56 pathogens, the pathogen prompt achieves mean top‐1 accuracy improvements of 20.1% and 17.6% over the pathogen average prompt and pathogen functional prompt. For the 10 most‐sampled pathogens, the pathogen prompt maintains a strong performance advantage, yielding mean top‐1 accuracy gains of 17.9% and 16.2% compared to the two alternatives. Notably, in the 10 least‐sampled pathogens, the pathogen prompt exhibits a marked performance boost, surpassing the other prompt types by 26.5% and 24.1%.

Additionally, we focused on the detailed performance of prompt guidance in low‐resource scenarios and conducted a t‐SNE visualization on peptides. As shown in Figure 3d, peptide representations without pre‐training reveal a disordered arrangement across least‐10 pathogens, failing to exhibit clear clustering among themselves (SC < 0). In contrast, peptide embeddings derived from pre‐training indicate enhanced clustering performance (SC = 0.235), reflecting our model's ability to encode meaningful peptide embeddings for various pathogens. Notably, in low‐resource scenarios, the accuracy of all guidance exhibits a decreasing trend as available peptides diminish (Figure 3e). Nevertheless, our generation with the pathogen prompt maintains robust generalizability, exhibiting lower variance in accuracy even for least‐10 pathogens, which highlights its reliability as an effective guidance, particularly in challenging cases where traditional approaches tend to falter. Furthermore, we randomly selected pathogens whose associated knowledge graph structures were retained for constructing pathogen‐specific prompts. Meanwhile, their corresponding peptide data were excluded from training the KPPepGen model to establish the zero‐shot scenario. As shown in Table S2 (Supporting Information), for the KPPepGen with zero‐shot setting, there is a notable decline in performance across both sequence‐ and structure‐level metrics compared to the KPPepGen model trained on the full dataset. Nevertheless, in comparison to generative models lacking conditional mechanisms (e.g., EvoDiff), KPPepGen demonstrates greater performance under the zero‐shot scenario (see details in Section S3.3, Supporting Information). These findings indicate that KPPepGen is capable of generating biologically plausible peptide sequences for previously unseen pathogens by leveraging knowledge pretraining and pathogen prompts, thereby highlighting the generalization ability of our model.

### 2.4 KPPepGen Captures Pathogen‐Specific Biological Patterns to Facilitate Peptide Generation

In this section, we explored the presence of essential biological patterns within the peptide data and evaluated KPPepGen's ability to capture these patterns to enhance peptide generation for specific pathogens.

**Figure 4:**
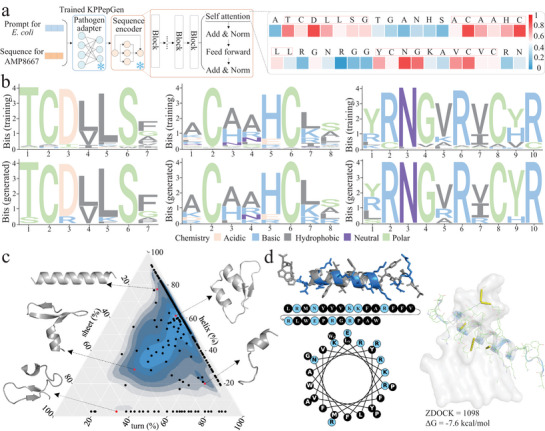
Identification of sequence and structure patterns for our generated peptides a) Schematic presentation of identifying pathogen‐related residue using the trained KPPepGen model. b) Sequence logos for three specific motifs present in training and generated peptides. c) The ternary diagram indicates the distribution of secondary structures. The scatter points represent generated peptides. The density gradient area displays the distribution of training peptides. d) Structure organization of the selected peptide. The helical wheel plot illustrates the arrangement of amino acids, highlighting hydrophobic residues in black and polar residues in blue. Docking posture visualization for this peptide with the general target for *E. coli* (PDB ID: 1QFG). The yellow lines indicate hydrogen bonds for side chains with the target.

First, we investigated pathogen‐related residue sites identified by the attention weights of KPPepGen, following the protocol described in the Methods section. As illustrated in **Figure** **4**a, KPPepGen identified a total of 23 high‐attention sites for the input sequence AMP8667. Next, we used *E. coli* peptides as examples and applied the STREME tool^[^ ^36^ ^]^ to identify the sequence motifs ranging from 5 to 15 residues in both the training and generated peptides, using a p‐value threshold of less than 0.05. As a result, 38 motifs were extracted from the training peptides and 31 from the generated peptides. We then employed the universalmotif tool^[^ ^37^ ^]^ to evaluate the similarity between pairwise motifs from the two sets. Figure 4b presents three of the top‐5 most similar motif pairs, which align with the key *E. coli*‐specific sites highlighted in Figure 4a (indicated by red boxes). The clear conservation of residues at critical positions, particularly those occupied by functionally important amino acids such as the positively charged arginine (R), neutral asparagine (N), and polar glycine (G), underscores the biological relevance of these conserved motifs in *E. coli*‐specific AMPs.

Furthermore, we employed the DSSP tool^[^ ^38^ ^]^ to extract secondary structure information from training and generated peptides, using *E. coli* as an example. As shown in Figure 4c, a ternary diagram illustrates the secondary structure composition of both peptide sets. The generated peptides exhibit varying degrees of helicity, particularly within the high‐density gradient region (shown in blue), indicating a structure composition comparable to that of the training peptides. Notably, five distinct conformational states (represented by gray peptides) underscore the structural diversity of the generated peptides, reflecting varied helix‐sheet‐turn compositions. In the bottom region of the ternary diagram, characterized by low helicity, the training peptides occupy a limited portion of the structure subspace. In contrast, the generated peptides are distributed across a broader area, exhibiting greater structural diversity under reduced helical constraints. Subsequently, we randomly selected a generated peptide from the high‐density gradient region, representing the predominant structure pattern. This peptide adopts a helical conformation, comprising ≈65% helical and 35% non‐helical content, and displays alternating clusters of hydrophobic and polar residues, as shown in the amino acid sequence and helical wheel plot (Figure 4d). This structure arrangement confers conformational flexibility, allowing the peptide to accommodate diverse structure features across different pathogen targets.^[^ ^39^, ^40^ ^]^ Docking analysis reveals a low binding energy of –7.6 kcal mol^−1^ and five hydrogen bonds against the general target for *E. coli* (PDB ID: 1QFG), along with a high ZDOCK score, indicating strong binding affinity. Similar structure patterns are observed in the generated peptides across multiple pathogens (see details in Section S3.4, Supporting Information). These findings demonstrate that KPPepGen effectively balances biological conservation and innovation, preserving the essential biological patterns while exploring the uncharted regions of the peptide space.

### 2.5 Peptide Optimization via the Extension of KPPepGen

In this section, we presented an extension of KPPepGen for peptide optimization, which integrates stochastic perturbation with iterative refinement to adjust peptide sequences toward improved properties.

**Figure 5:**
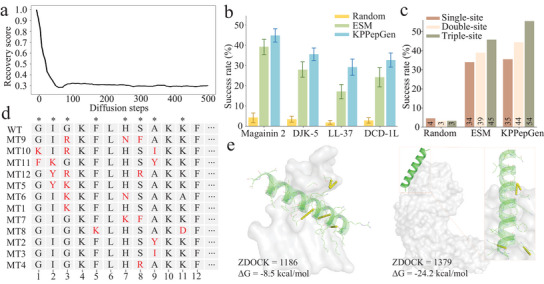
Extension of KPPepGen for peptide optimization. a) Recovery scores quantifying sequence similarity between wild‐type peptide and mutants, plotted within diffusion steps. b) Success rates of AMP mutants yielded by different methods. c) Success rates of Magainin 2 mutants yielded by different methods, grouped by the number of mutation sites. d) 12 mutants of the wild‐type (WT) Magainin 2 ranked by their optimized performance, with mutations highlighted in red. Mutation type (MT) peptides MT1 to MT4 correspond to single‐site mutants, MT5 to MT8 represent double‐site mutants, and MT9 to MT12 contain triple‐site mutants. The symbol '*' indicates high‐attention sites previously identified by KPPepGen. e) Conformations of the top‐performing mutant (MT9) against the general (left, PDB ID: 1QFG) and specific (right, PDB ID: 3MZE) targets of *E. coli*. The yellow lines indicate hydrogen bonds for side chains with targets.

First, to initiate the optimization protocol of KPPepGen (Figure 1e), we subjected the wile‐type peptides to the stochastic perturbation over *t* iterative steps using a forward diffusion process, while the denoising phase commenced from the (*T* − *t*)‐th step (corresponding to *t* remaining steps) within the diffusion model, enabling the reconstruction of a set of mutants (see details in the Methods section). As shown in **Figure** **5**a, the mutants undergo the progressive exploration of novel sequence space as diffusion steps increase (represented by decreasing recovery score), eventually stabilizing around a score of 0.3 at 100 steps, which was selected as the diffusion parameter for peptide optimization.

Following this optimization protocol, two natural AMPs (Magainin 2^[^ ^41^ ^]^ and DJK‐5^[^ ^42^ ^]^) and two human AMPs (LL‐37^[^ ^43^ ^]^ and DCD‐1L^[^ ^44^ ^]^) were subjected to 100 mutants at one, two, or three high‐attention sites identified by KPPepGen for the multi‐site combinatorial optimization, resulting in 300 mutants per AMPs. A mutant was considered to be successfully optimized if it exhibited lower instability and TM_tend values, as well as higher docking scores against pathogen‐specific targets, compared to the wild‐type peptide. The success rate is defined as the proportion of successfully optimized mutants. For comparison, we also included the ESM‐based optimization method^[^ ^45^ ^]^ and the random mutation strategy as baselines. As shown in Figure 5b, KPPepGen achieved the highest success rate across four AMPs (e.g., 44.3% for Magainin 2), with an average improvement of 7.6% over the ESM‐based method, highlighting the broad applicability of our model to peptides derived from diverse biological sources. In contrast, the random mutation strategy consistently produced the lowest success rate, falling below 5%. We further evaluated the performance of these three methods using Magainin 2 as a representative example, across single‐, double‐, and triple‐site mutation settings (Figure 5c). The random mutation strategy shows the lowest success rate across all settings. For single‐site mutations, KPPepGen and the ESM‐based method demonstrate comparable results, with only a 1% difference. However, as the number of mutation sites increased, KPPepGen demonstrated clear advantages, with improvements of 5% and 9% for double‐ and triple‐site mutations.

Furthermore, we selected the top four Magainin 2 mutants (based on the average of their ranks across instability, TM_tend values, and docking scores), for each of the corresponding single‐, double‐, or triple‐site mutations, and then re‐ranked them. As shown in Figure 5d, triple‐site mutants indicate the best overall performance, followed by double‐site mutants, and single‐site mutants performed the worst. Then, we analyzed mutant MT9, the best‐performing mutant, by examining its docking conformations against both general and specific targets of *E. coli*. As shown in Figure 5e, MT9 exhibits low docking energies for the general target (–8.5 kcal mol^−1^, PDB ID: 1QFG) and specific target (–24.2 kcal mol^−1^, PDB ID: 3MZE), along with high ZDOCK scores. Notably, the structure analysis reveals well‐defined interactions with the polar regions of the target surface, including five and four hydrogen bonds with the general and specific targets, respectively. These findings underscore the capability of KPPepGen to explore high‐dimensional sequence space and effectively guide the design of complex multi‐site mutations, thereby generating peptides with enhanced properties. Notably, the peptide optimization is not limited to existing AMPs but can also be applied to our generated peptides, enabling further improvement in their potential for practical applications.

### 2.6 Wet‐Lab Evaluation of Peptides Generated by KPPepGen Against *E. coli* and *S. aureus*

Herein, we focused on the generated peptides targeting two clinically significant pathogens, *E. coli* and *S. aureus*, which are of particular importance due to their widespread involvement in infectious diseases.^[^ ^46^ ^]^

**Figure 6:**
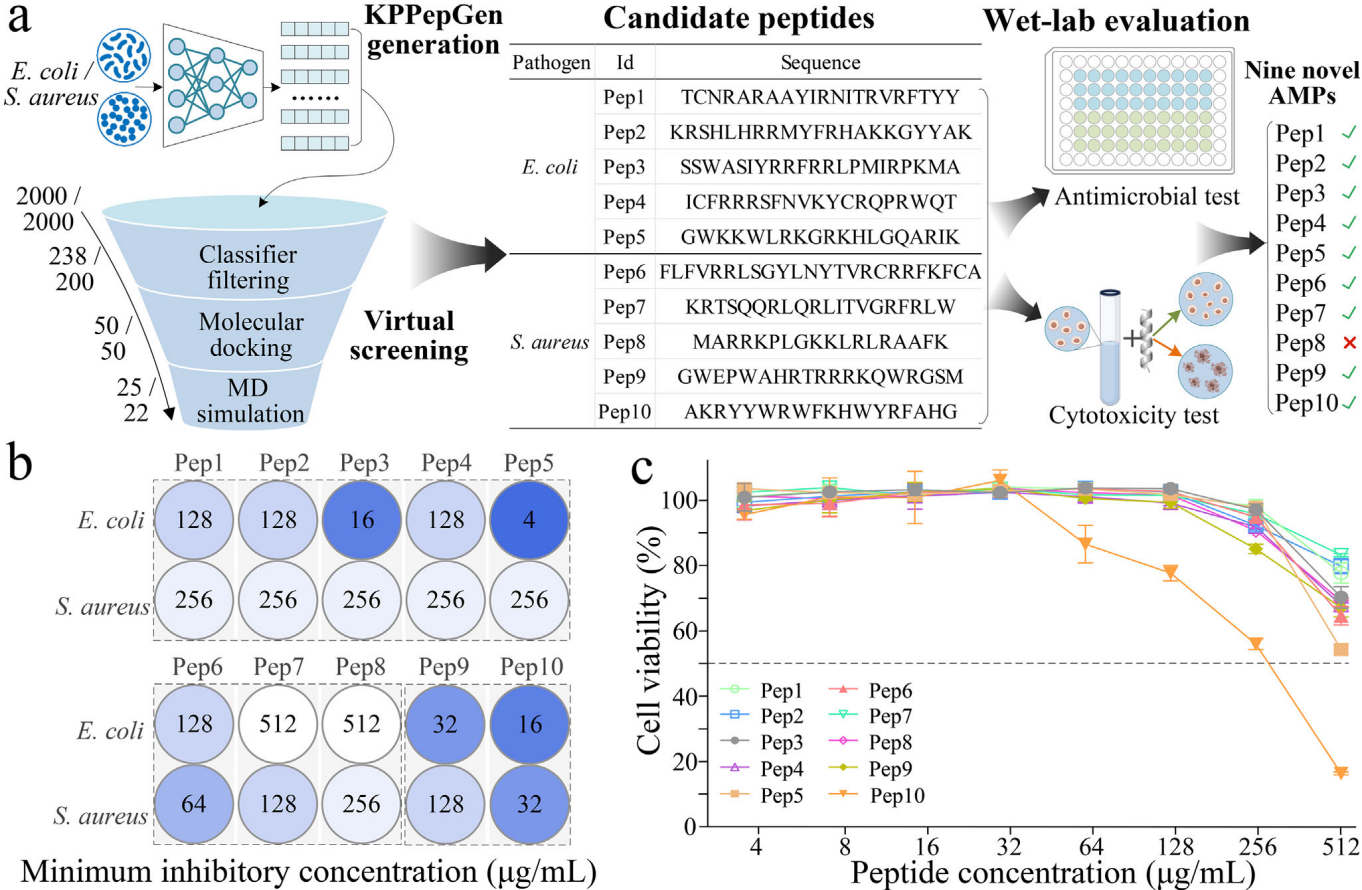
Virtual screening of peptides and evaluation of antimicrobial activity and cytotoxicity. a) Flowchart of the virtual screening for candidate peptides targeting *E. coli* and *S. aureus*. b) MIC of these peptides assessing against *E. coli* and *S. aureus*. c) The cytotoxicity of these peptides is evaluated using the CCK‐8 assay across various concentrations. The dotted line indicates 50% cell viability.

First, we employed the well‐trained KPPepGen to generate 2000 peptides for *E. coli* and *S. aureus*, respectively. We then introduced a virtual screening procedure to prioritize the most promising candidates for wet‐lab evaluation (see details in Section S2.2, Supporting Information). As shown in **Figure** **6**a, peptides with predicted antimicrobial efficacy above a confidence threshold of 0.95 were first selected using pathogen‐specific classifiers for *E. coli* and *S. aureus*. These high‐confidence peptides were further screened based on molecular docking scores against the corresponding pathogen‐specific targets. Subsequently, molecular dynamics (MD) simulations were performed for these peptide–target complexes. Binding free energy was estimated using the Molecular Mechanics Poisson–Boltzmann Surface Area (MM‐PBSA) method,^[^ ^47^ ^]^ and conformational stability was evaluated via root‐mean‐square‐fluctuation (RMSF) analyses from simulation trajectories. Following this virtual screening procedure, we identified 5 candidate peptides for *E. coli* and *S. aureus*, respectively (Figure 6a).

Indeed, a more conservative activity threshold for AMPs should be considered clinically significant.^[^ ^19^ ^]^ Following the previous studies,^[^ ^19^, ^46^ ^]^ we defined the antimicrobial activity threshold as minimum inhibitory concentrations (MIC) ⩽ 128 µg mL^−1^. As shown in Figure 6b, using the above activity threshold, 9 out of 10 candidate peptides are confirmed as effective AMPs, reflecting a 90% success rate. Notably, three AMPs demonstrated high levels of antimicrobial activity for specific pathogens (MIC ⩽ 32 µg mL^−1^), including Pep3 (16 µg mL^−1^) and Pep5 (4 µg mL^−1^) against *E. coli*, as well as Pep10 (16 µg mL^−1^) against *S. aureus*. To further evaluate the antimicrobial specificity for the given pathogen, we retested these candidates for antimicrobial activity against the other pathogen. Notably, all five candidates (Pep1 to Pep5) against *E. coli* exhibit low activity (MIC ⩾ 256 µg mL^−1^) against *S. aureus*. Among three candidates (Pep6 to Pep8) initially designed for *S. aureus*, Pep7 and Pep8 exhibited no detectable activity (MIC ⩾ 512 µg mL^−1^) against *E. coli*, yet only Pep6 demonstrated the activity against *E. coli*. We also validated that two candidates (i.e., Pep9 and Pep10) exhibit antimicrobial activity against both pathogens. Additionally, we assessed the cytotoxicity of these candidates by determining the peptide concentration causing 50% cytotoxicity (CC50). It is generally observed that enhancements in antimicrobial activity are often accompanied by increased cytotoxicity.^[^ ^48^ ^]^ As shown in Figure 6c, we displayed the cell viability of candidates across the concentration gradient. Notably, 9 out of 10 candidates exhibit completely non‐toxic (CC50 ⩾ 512 µg mL^−1^) and only Pep10 demonstrates slightly low toxicity (CC50 ⩾ 256 µg mL^−1^), highlighting the strong antimicrobial efficacy and low to negligible cytotoxicity of generated peptides. Consequently, these findings confirm the outstanding generative performance of KPPepGen, which successfully generates peptides tailored to given pathogen requirements, achieving both strong antimicrobial efficacy and favorable cytotoxicity property.

## 3 Conclusion

In this study, we introduce KPPepGen, a knowledge‐aware prompt diffusion model for the controllable generation of pathogen‐specific antimicrobial peptides. KPPepGen provides several notable advantages, as outlined below: 1) Compared to existing state‐of‐the‐art methods, KPPepGen is capable of simultaneously generating peptides for 56 distinct pathogens, demonstrating superior performance with favorable properties and docking efficacy across a wide range of pathogen targets. 2) Comprehensive analysis of the pathogen prompts highlights their pivotal role in facilitating the generation of pathogen‐specific peptides, effectively enabling the identification of essential sequence and structure patterns tailored to specific pathogens. Moreover, the peptide optimization extended from KPPepGen further enhances the diverse properties of peptides, thereby improving their potential for practical applications. 3) The wet‐lab evaluation confirmed that nine novel antimicrobial peptides generated by KPPepGen are effective for the given pathogen requirements, exhibiting potent antimicrobial efficacy alongside favorable cytotoxicity property. Given these encouraging outcomes, KPPepGen signifies a promising advancement in the challenging task of designing novel, pathogen‐specific AMPs with therapeutic potential.

Notably, the core contribution of our model lies in leveraging biological knowledge of peptides to construct pathogen‐specific prompts that guide the generative process. Although we employ the Denoising Diffusion Implicit Models (DDIM) framework to accelerate the sampling process, the model's training and inference efficiency remain constrained by the limitations inherent to conventional diffusion models. Given the advantages of flow‐matching models, particularly in terms of faster training and more efficient sampling, this class of models has garnered increasing attention in drug discovery.^[^ ^49^ ^]^ In future work, we aim to explore the integration of advanced flow‐matching models to further improve the efficiency and performance of peptide generation.

## 4 Experimental Section

In this study, the construction of GO and pathogen knowledge graphs for peptide data was first outlined. Then, a detailed description of the knowledge‐aware pre‐training process, with particular emphasis on the learned pathogen embeddings, which served as prompts for their respective pathogens, is provided. Subsequently, the architecture of the prompt‐guided peptide diffusion framework was examined, with a particular emphasis on the role of prompt guidance during both the noising and denoising process. Additionally, the extended application of KPPepGen in identifying pathogen‐related residue sites and optimizing peptides, was elaborated. Finally, the peptide datasets, baselines, evaluation metrics, and wet‐lab evaluation utilized to assess generation performance were introduced.

### Construction of GO and Pathogen Knowledge Graphs

By leveraging peptide data from the UniProt database and AMP datasets described in the Datasets section, the GO and pathogen‐specific knowledge graphs (Figure 1b) were constructed. Specifically, entities *e*_*GO*_ and *e*_*pathogen*_ were denoted as class nodes, characterized by the annotation texts for GO and pathogen terms. The entity *e*_*peptide*_ represented the peptide node, characterized by the residue sequence with the corresponding amino acid type, which was associated with class nodes. Furthermore, a triple of the knowledge graph was defined as (*h*, *r*, *t*), where *h* and *t* were the head and tail entities, and *r* was the relation. The triples in GO and pathogen knowledge graphs can be separately categorized into two distinct types: *triple*_*GO*2*GO*_ and *triple*_*pathogen*2*pathogen*_, which represented the hierarchical associations among class nodes, as well as *triple*_*peptide*2*GO*_ and *triple*_*peptide*2*pathogen*_, which denoted the relationships between peptide and class nodes.

### Knowledge‐Aware Pre‐Training for Pathogen Prompts

In this section, the aim was to learn the embeddings for both class and peptide nodes from the GO and pathogen knowledge graphs, enabling the integration and understanding of complex biological information.

First, the PubMedBERT^[^ ^50^ ^]^ was employed to process the annotation texts corresponding to entities *e*_*GO*_ and *e*_*pathogen*_ and extracted their representations, denoted as *H*_*GO*_ and *H*_*pathogen*_, through averaging the features of all tokens within the annotation text. Then, a sequence encoder based on the transformer architecture was designed to process the amino acid sequence of entity *e*_*peptide*_, and extracted the corresponding representation, denoted as *H*_*peptide*_, by averaging features of all the amino acids. Notably, to bridge the gap between entities *e*_*GO*_ and *e*_*pathogen*_ and entity *e*_*peptide*_, the affine transformation (extra trainable linear layers) was utilized to project their representations to a unified dimensional space.

Subsequently, a contrastive learning strategy augmented with knowledge‐aware negative sampling^[^ ^25^, ^28^ ^]^ for the pre‐training process, was implemented. Specifically, peptide nodes were randomly sampled and retrieved their corresponding class nodes to construct subgraphs for GO or pathogen knowledge. In the subgraph, the positive triple (*h*, *r*, *t*) utilized *H*_*peptide*_, *H*_*GO*_ or *H*_*pathogen*_ as the node representation for the head and tail entities, along with the pre‐defined one‐hot features representing the relation. Then, (*h*, *r*, *t*′) denoted the corresponding negative triple through the triple augmentation, where the tail entity *t*′ was selected from the GO or pathogen knowledge. For instance, if the positive case is *triple*_*peptide*2*pathogen*_, the negative case retains the head entity (e.g., a peptide instance), and replaces the tail entity with an unrelated entity (e.g., another pathogen class). Formally, the objective of the knowledge‐aware pre‐training process was defined as follows:

(1) $$L_{triple}=-\log\sigma \left(\gamma -d\left(h,r,t\right)\right)-\log\sigma \left(d\left(h,r,t^{\prime }\right)-\gamma \right)$$

where σ was the sigmoid function, and γ represents the margin value. The function *d* served as the scoring metric to maximize the distinction between positive and negative triples, and the TransE^[^ ^51^ ^]^ was used for this calculation,

(2) d(h,r,t)=∥h+r−t∥

The model was sequentially pre‐trained on the GO and pathogen knowledge graphs and subsequently extracted the learned embedding *C* of pathogen terms from the pathogen knowledge graph, defining them as knowledge injections in the form of pathogen prompts to guide the peptide diffusion model. Additionally, the pre‐trained sequence encoder was made available for further analysis. To incorporate new peptide–pathogen relationships, pathogen names should be standardized according to the nomenclature provided in the gcPathogen database.^[^ ^21^ ^]^ The new entries should be formatted as triples and added to the specific file. The pretrained knowledge model can be fine‐tuned to update its parameters accordingly. More details can be found in Section S2.1 (Supporting Information).

### Prompt‐Guided Peptide Diffusion Generation

For peptide sequences, the residue types were treated as categorical data and applied the discrete diffusion process to the sequences. In the forward process, residue types were encoded using one‐hot encoding, and the diffusion noise was represented by transition matrices (**Q** ^1^, **Q** ^2^, …, **Q** ^*T*^), where [**Q** ^*t*^]_*ij*_ signifies the corresponding probability of jumping from residue type *i* to type *j* at timestep t: *q*(*S* ^*t*^|*S* ^*t* − 1^) = *S* ^*t* − 1^ **Q** ^*t*^. Herein, the marginal transitions^[^ ^52^ ^]^ parameterized by:

(3) $$\;\;\;\;Q^{t}=\alpha^{t}I+\left(1-\alpha^{t}\right)1_{i}m_{S}$$

where α^*t*^ transitions from 1 to 0, were employed. The transition matrix from *S* ^0^ to *S* ^*t*^ was represented as ${\bar{Q}}^{t}=Q^{1}Q^{2}\text{…}Q^{t}$, and the noisy statue *S* ^*t*^ could be defined as $q\left(S^{t}|S^{0}\right)=S^{0}{\bar{Q}}^{t}$. Additionally, *m*_*S*_ was distinct from the uniform transition and represented the marginal distribution^[^ ^53^ ^]^ for residue type *i* in the peptide dataset, i.e., $\forall i,\lim_{T\to \infty }{\bar{Q}}^{T}1_{i}=m_{S}$. The probability *q*(*S* ^*t* − 1^|*S* ^*t*^, *S* ^0^) was computed with the Bayes rule:^[^ ^53^ ^]^

(4) $$\;\;q\left(S^{t-1}|S^{t},S^{0}\right)\propto S^{t}{\left(Q^{t}\right)}^{\prime }⊙S^{0}{\bar{Q}}^{t-1}$$

where ⊙ denotes a pointwise product and **Q**′ is the transpose of **Q**. In the reverse process, the diffusion trajectory was parameterized by the probability *q*(*S* ^*t* − 1^∣*S* ^*t*^, *S* ^0^),^[^ ^54^ ^]^ while a network ${\hat{p}}_{\theta }$ was defined to predict the probability of *S* ^0^,

(5) $$p_{\theta }\left(S^{t-1}|S^{t}\right)=\prod_{1\leq i\leq N}q\left(s_{i}^{t-1}|S^{t},{\hat{S}}^{0}\right)\cdot {\hat{p}}_{\theta }\left({\hat{S}}^{0}|S^{t}\right)$$

where $s_{i}^{t}$ denotes the embedding of the *i*‐th residue for the peptide sequence *S* at timestep *t*, and ${\hat{S}}^{0}$ is the predicted probability of *S* ^0^, *N* represents the number of residues in the peptide sequence.

Subsequently, the classifier‐free guidance (CFG) strategy^[^ ^55^ ^]^ was employed to enhance the influence of prompt guidance for the diffusion process. The loss function for training this process was the mean squared error (MSE)^[^ ^56^ ^]^ between the predicted noise of ϵ_θ_ and priori noise ϵ.

(6) $$\;L\left(\theta \right)=E_{t∼\text{Uniform}(1,T)}{∥\varepsilon -\varepsilon_{\theta }\left(z_{t},t,C\right)∥}_{2}^{2}$$

where the Uniform(1…T) shows the uniform distribution for the diffusion timesteps. Notably, CFG is formulated as ωϵ_*c*_ + (1 − ω)ϵ_*u*_, where ω, ϵ_*c*_, ϵ_*u*_ are the CFG weight, conditional output, and unconditional output. In the reverse process, the null‐text prompt for the unconditional prediction lacked a valid pathogen type to describe it. Herein, the general pathogen $\bar{C}$ was defined as the null‐text prompt, which was the average of all the pathogen prompt embeddings. Then, prompt $\bar{C}$ was utilized for the reverse process^[^ ^57^ ^]^:

(7) $${\hat{\varepsilon }}_{\theta }=\varepsilon_{\theta }\left(z_{t},t,\bar{C}\right)+\omega \left(\varepsilon_{\theta }\left(z_{t},t,C\right)-\varepsilon_{\theta }\left(z_{t},t,\bar{C}\right)\right)$$

where the guidance scale ω controls the strength of the conditional prediction ϵ_*c*_ = ϵ_θ_(*z*_*t*_, *t*, *C*) and unconditional prediction $\varepsilon_{u}=\varepsilon_{\theta }\left(z_{t},t,\bar{C}\right)$.

Furthermore, pathogen prompts, along with a pathogen adapter, were employed to explicitly guide the peptide feature space, aligning it with the data distribution of specific pathogens. Unlike conventional noise priors such as uniform distributions, pathogen‐specific marginal distributions were introduced as the prior within the diffusion model. Specifically, the frequency distributions of 20 standard amino acids were quantified within the peptide data for each pathogen, as well as across all AMPs, which represent the marginal distribution in Equation (3), including $m_{S}^{c}$ for the pathogen prompt in conditional prediction and $m_{S}^{u}$ for the general prompt in unconditional prediction. Through pretraining in knowledge graphs, the diffusion model learns to effectively control the distribution of peptide features, as well as pathogen‐specific prior, which enhances the model's ability to generate peptides that are appropriately tailored to different pathogens. Therefore, combining Equations (5) and (7), we formulated the ${\hat{p}}_{\theta }$ as follows:

(8) $${\hat{p}}_{\theta }\left({\hat{S}}^{0}|S^{t},C,\bar{C}\right)=\prod_{1\leq i\leq N}\text{Softmax}\left({\hat{s}}_{i}^{0}|{\hat{\varepsilon }}_{\theta }\left(h_{i}^{t},t,C,\bar{C}\right)\right)$$

where $h_{i}^{t}$ is the embedding of residue *i* for peptide sequence *S* at timestep *t*. Additionally, ${\hat{\varepsilon }}_{\theta }$ was a neural network framework to predict the noise of residue types guided by the pathogen prompts, and then the noise would be removed to compute the probability of ${\hat{s}}_{i}^{0}$. The Softmax function was applied over all residues of the input peptide sequence *S*. Herein, the objective of the prompt‐guided diffusion for pathogen‐specific peptide generation was to minimize the expected Kullback‐Leibler (KL) divergence between the posterior and predicted distribution of residue types^[^ ^58^, ^59^ ^]^ at each timestep *t*:

(9) $$L_{S}^{t}=\frac{1}{N}\sum_{1\leq i\leq N}D_{KL}\left(q\left(s_{i}^{t-1}|S^{t},S^{0}\right)∥p_{\theta }\left({\hat{s}}_{i}^{t-1}|S^{t},C,\bar{C}\right)\right)$$

Furthermore, the final training objective function of the peptide diffusion process was denoted as:

(10) $$\;\;\;L_{S}=E_{t∼\mathrm{Uniform}(1\text{…}T)}\left[L_{S}^{t}\right]$$

### Extended Application of KPPepGen

Herein, the extension of KPPepGen aimed to identify pathogen‐related residue sites and optimize peptides, was introduced.

To identify pathogen‐related residue sites, the well‐trained KPPepGen model, freezing the parameters of both the pathogen adapter and sequence encoder, and used the peptide sequence along with the corresponding pathogen prompt as input (Figure 4b), was leveraged. Then, the attention weights of residue sites was extracted from the final transformer attention layer of the sequence encoder. These weights were employed to calculate normalized importance values, quantifying the contribution of individual residue sites to noise predictions under prompt guidance. Residue sites with importance values surpassing a pre‐defined threshold (i.e., 0.45) were identified as sites for specific pathogens.

To initiate the optimization protocol, the wild‐type peptide sequences was subjected to stochastic perturbation with the pathogen prompt over *t* iterative steps using a forward diffusion process. The denoising phase commences from the (*T* − *t*)‐th step (corresponding to remaining steps) within the diffusion model, enabling the reconstruction of a set of candidate peptides. Subsequently, the pathogen‐related residue sites was identified within the wild‐type peptide sequence as mutation targets and randomly introduced site‐specific mutations at one, two, or three positions. A noising‐denoising process was then applied to these sites to produce a diverse set of mutants. Furthermore, mutants in which the mutated sites exhibited higher likelihood estimates than their corresponding positions in the wild‐type sequence, considering them as candidates for optimization, was selected.

### Experimental Setting

**Datasets**: Leveraging the UniProt database,^[^ ^60^ ^]^ a peptide dataset, encompassing a diverse collection of peptide sequences derived from various organisms, was constructed. All peptides have lengths from 5 to 50 amino acids and exclusively consist of 20 standard amino acid types. Sequence redundancy was minimized using the CD‐HIT tool by removing sequences with ⩾ 95% similarity. Specifically, over 764 000 peptide sequences annotated with GO terms were collected, covering 7266 distinct GO categories, and a total of 1.36 million peptide‐GO pairs were extracted. Each GO term was accompanied by a textual description retrieved from the GO‐basic file available on the Gene Ontology website. Subsequently, antimicrobial peptides are derived from a manually curated peptide dataset, combining experimentally validated peptides from seven known AMP databases, including APD3,^[^ ^61^ ^]^ CAMP,^[^ ^62^ ^]^ DBAMP,^[^ ^63^ ^]^ DRAMP,^[^ ^64^ ^]^ SATPdb,^[^ ^65^ ^]^ YADAMP,^[^ ^66^ ^]^ and LAMP.^[^ ^67^ ^]^ From these sources, relationships indicating the inhibitory activity of peptides against specific strains of pathogens, were extracted. To unify and standardize the strain information, strain annotations were merged into pathogen‐level labels using the gcPathogen database^[^ ^21^ ^]^ and manually validated pathogen names for consistency. Then, a total of 14 110 AMPs annotated with specific pathogen labels and 64 487 peptide‐pathogen pairs, spanning 56 distinct pathogen types, were collected. Each pathogen includes a textual description from PubMed and Wikipedia websites. More details about datasets can be found in Section S1.1 (Supporting Information).

**Baselines**: For the peptide generation, the baseline methods were summarized into four distinct types, i.e., positive‐only learning, discriminator‐guided filtering approaches, latent space sampling, and conditional generation approaches. Specifically, for the positive‐only learning approaches, LSTM‐RNN,^[^ ^68^ ^]^ AMPGen,^[^ ^13^ ^]^ ProtGPT2,^[^ ^69^ ^]^ and EvoDiff^[^ ^70^ ^]^ were utilized as baselines. For the discriminator‐guided filtering approaches, AMPTrans,^[^ ^14^ ^]^ LSTM‐Pep,^[^ ^71^ ^]^ AMPGAN,^[^ ^16^ ^]^ and RLGen^[^ ^15^ ^]^ were employed as baselines. For the latent space sampling approaches, WAE‐PSO,^[^ ^18^ ^]^ ProteoGAN,^[^ ^72^ ^]^ and AMPGAN‐v2^[^ ^73^ ^]^ were selected as baselines. For the conditional generation approaches, ProGen,^[^ ^74^ ^]^ HydrAMP,^[^ ^19^ ^]^ AR‐VAE,^[^ ^75^ ^]^ Cut&CLIP,^[^ ^28^ ^]^ and PrefixProt^[^ ^76^ ^]^ were employed as baselines. Notably, ten of these models were originally designed specifically for peptide generation: LSTM‐RNN, AMPGen, AMPTrans, LSTM‐Pep, AMPGAN, RLGen, WAE‐PSO, AMPGAN‐v2, HydrAMP, and Cut&CLIP. Meanwhile, six models, including ProtGPT2, EvoDiff, ProteoGAN, ProGen, AR‐VAE, and PrefixProt, were initially developed for protein generation and subsequently adapted for peptide generation tasks. More details about baselines can be found in Section S1.2 (Supporting Information).

**Evaluation Metrics**: The evaluation of generated peptides was conducted using the following metrics. For peptide sequences, the similarity score, calculated using the Needleman–Wunsch algorithm from the Biopython package, serves as a metric to assess the resemblance between training and generated sequences. The low similarity score indicates a high level of novelty in generated sequences. The instability score was used to evaluate the degree of peptide instability, relying on the composition of amino acids within the sequence. The TM_tend score^[^ ^77^ ^]^ indicates the amino acid transmembrane propensity scale, offering insights into the bacteriostatic capacity of peptides to a certain extent. Subsequently, the physicochemical properties of peptides, including charge, isoelectric point, hydrophobic, and aromaticity, were evaluated. The instability and TM_tend scores, along with physicochemical properties were assessed using the modlAMP^[^ ^17^ ^]^ tool. Herein, the Student's t‐test was employed to assess differences in the distribution of indicators between the training and generated data. To control for multiple hypothesis testing, p‐values were adjusted using the Benjamini–Hochberg procedure. Statistical significance was defined as an adjusted p‐value less than 0.05. The trRosetta tool^[^ ^35^ ^]^ was employed to predict peptide structures and utilized ZDOCK and AutoDock Vina tools^[^ ^78^ ^]^ to assess docking scores of peptides to their pathogen targets. Specifically, the general targets of pathogens were selected based on structural data from the Protein Data Bank (PDB) for different categories: Gram‐positive pathogens (PDB ID: 6YFY), Gram‐negative pathogens (PDB ID: 1QFG), and Fungus pathogens (PDB ID: 1ZHS). Notably, specific targets are available in the PDB for only 22 out of the 56 pathogens considered in this study, including E. coli (3MZE), S. aureus (5M18), A. baumannii (3UDI), E. faecalis (3E6E), P. aeruginosa (3OC2), K. pneumoniae (8GPW), L. monocytogenes (3ZG8), B. cereus (7BN9), S. pneumoniae (5OJ1), M. tuberculosis (6KGH), B. subtilis (7BN9), S. epidermidis (8C5B), E. faecium (6BSQ), L. innocua (3ZG7), B. licheniformis (1NRF), S. typhimurium (4Q6L), E. cloacae (5XHR), S. enterica (4Q6V), C. albicans (7STO), S. cerevisiae (8K3Q), A. fumigatus (2XVN), and A. niger (6IGY). More details about metrics can be found in Section S1.3 (Supporting Information).

**Wet‐Lab Evaluation**. All peptides were synthesized using the Fmoc‐based solid‐phase peptide synthesis (SPPS) method, with a purity greater than 98%, by Nanjing GenScript Biotechnology Co., Ltd. More details about the wet‐lab evaluation can be found in Section S2.3 (Supporting Information).

## Conflict of Interest

The authors declare no conflict of interest.

## Supporting information

Supporting Information

## Data Availability

All training data utilized in this study were obtained from publicly accessible resources. Gene Ontology annotations for peptides, available through the GO‐basic file, can be found at https://www.geneontology.org. Peptide data with GO relationships are sourced from the UniProt database at https://www.uniprot.org/uniprotkb. Antimicrobial peptide databases used include APD3: https://aps.unmc.edu/, CAMP: https://camp3.bicnirrh.res.in, DBAMP: https://awi.cuhk.edu.cn/dbAMP, DRAMP: http://dramp.cpu‐bioinfor.org, SATPdb: http://crdd.osdd.net/raghava/satpdb, YADAMP: http://www.yadamp.unisa.it, and LAMP: http://biotechlab.fudan.edu.cn/database/lamp. The pre‐processed GO/pathogen knowledge data and the peptide sequence data employed in this study are available at https://zenodo.org/records/15660801. The source code of this study is freely available at https://github.com/wyky481l/KPPepGen.
