# Supplementary material for: Controllable Generation of Pathogen‐Specific Antimicrobial Peptides Through Knowledge‐Aware Prompt Diffusion Model
Source: Adv Sci (Weinh). 2025 Sep 15;12(41):e07457. doi: 10.1002/advs.202507457 (PMC12591163; doi:10.1002/advs.202507457)
Supplement: Supplementary file 1 — Supporting Information [file ADVS-12-e07457-s001.pdf]

# Supplementary information

## Contents

**1 Experimental setups** **1**
1.1 Details of the datasets . . . 1
1.2 Details of the baseline methods . . . 1
1.3 Details of the evaluation metrics . . . 2
1.4 Details of the implementation . . . 3
**2 Methods** **4**
2.1 Details of the knowledge-aware pre-training . . . 4
2.2 Details of the virtual screening procedure . . . 4
2.3 Details of the wet-lab evaluation . . . 5
**3 Results** **5**
3.1 More landscape for peptide datasets . . . 5
3.2 More performance for generation with pathogen prompts . . . 6
3.3 Peptide generation under the zero-shot scenario . . . 6
3.4 More pattern exploration for generated peptides . . . 7
3.5 Ablation study . . . 9
3.6 Peptide generation under various length ranges . . . 9
**References** **10**

## 1 Experimental setups

### 1.1 Details of the datasets

In this study, all peptide sequences range in length from 5 to 50 amino acids and exclusively consist of 20 standard amino acids. Sequence redundancy was minimized using the CD-HIT tool by removing sequences with $\geqslant 95\%$ similarity. Subsequently, Gene Ontology (GO) annotations corresponding to the peptide sequences were retrieved from the UniProt database. Peptides with fewer than three associated GO terms were excluded to ensure sufficient functional information. Then, we collected over 764,000 peptide sequences annotated with GO terms, covering 7,266 distinct GO categories, and extracted a total of 1.36 million peptide-GO pairs. Each GO term is accompanied by a textual description retrieved from the GO-basic file available on the Gene Ontology website, and we extracted a total of 42,837 GO terms and 83,581 GO-GO triples. These GO terms cover three aspects: biological process, cellular component, and molecular function. Each GO term includes a text-annotated description.

Subsequently, we derived antimicrobial peptides (AMPs) from the manually curated AMP datasets, combining experimentally validated peptides from 7 known databases, including APD3[1], CAMP[2], DBAMP[3], DRAMP[4], SATPdb[5], YADAMP[6], and LAMP[7]. From these AMP sources, we extracted relationships indicating the inhibitory activity of peptides against specific strains of particular pathogens. To unify and standardize the strain information, we merged strain annotations into pathogen-level labels using the gcPathogen database[8] and manually validated pathogen names for consistency. Then, we collected a total of 14,110 AMPs annotated with specific pathogen labels and 64,487 peptide-pathogen pairs, spanning 56 distinct pathogen types, involving the Gram-positive/Gram-negative bacteria and Fungus. Each pathogen includes a textual description from the PubMed and Wikipedia websites. Notably, among 56 pathogen types examined in this study, only 17 pathogens (30% of the cases) have over 500 AMPs, while 42 pathogens (75% of the cases) have more than 200 AMPs. Additionally, 14 pathogens (the remaining 25% of the cases) possess peptide counts ranging from 100 to 200. From the UniProt database, we further searched peptides excluding the keywords 'antimicrobial, antibiotic, antiviral, and antifungal', and applied the CD-HIT tool to remove sequences with repeatability over the 0.6 threshold, which resulted in a non-AMP dataset containing 18,458 peptides without the antimicrobial efficacy. The pre-processed GO/strain knowledge data and peptide sequence data utilized in our KPPepGen study are available at our Zenodo repository.

### 1.2 Details of the baseline methods

For the controllable peptide generation, we comprehensively evaluated the performance in comparison with existing peptide generative methods. These approaches could be categorized into four distinct types, i.e., positive-only learning, discriminator-guided filtering, latent space sampling, and conditional generation approaches. Specifically, positive-only learning methods rely on the underlying distribution of the training data to generate peptides, requiring the training of individual generative models

for each pathogen (i.e., one-against-one strategy). In contrast, discriminator-guided filtering approaches utilize predefined screening criteria to produce pathogen-specific peptides. These approaches involve training generative models on peptides from all pathogens, followed by the application of pathogen classifiers to filter peptides for individual pathogens. Latent space sampling and conditional generation approaches integrate the additional constraint information into the generative process, such as property labels and pathogen classifiers.

Herein, we employed the autoregression-based, generative adversarial network-based, variational autoencoder-based, and diffusion-based models as baselines across various generative frameworks. Specifically, the positive-only learning approaches involve capturing the distribution of a peptide dataset for a given pathogen type and subsequently sampling from this distribution to generate new peptides, such as LSTM-RNN[9], AMPGen[10], ProtGPT2[11], and EvoDiff[12]. Leveraging the advancements in pre-training language models (PLMs)[13], we utilized the PLM-based method ProtGPT2[11] as the baseline, which is pre-trained on large-scale protein data. Additionally, we extended EvoDiff[12], a diffusion-based method originally developed for proteins. Then, the discriminator-guided filtering approaches involve training a peptide generator using mixed peptide data (e.g., AMPs and non-AMPs), which compete to produce new peptides, followed by the additional evaluation tools to filter out inactive sequences, such as AMPTrans[14], LSTM-Pep[15], AMPGAN[16], and RLGen[17]. Additionally, the latent space sampling approaches integrate additional constraint information, such as property labels and antimicrobial discriminators, allowing them to guide the peptide distribution relevant to given constraints, including WAE-PSO[18], ProteoGAN[19], and AMPGAN-v2[20]. Also, the conditional generation approaches involve learning from various peptide datasets while employing the condition information to design peptide sequences that meet specific criteria, including ProGen[11], HydrAMP[21], AR-VAE[22], Cut&CLIP[23], and PrefixProt[24]. Notably, 10 of these models were designed specifically for peptide generation: LSTM-RNN, AMPGen, AMPTrans, LSTM-Pep, AMPGAN, RLGen, WAE-PSO, AMPGAN-v2, HydrAMP, and PepCLIP. Meanwhile, 6 models, including ProtGPT2, EvoDiff, ProteoGAN, ProGen, AR-VAE, and PrefixProt, were initially developed for protein generation and subsequently adapted for peptide generation tasks.

Specifically, LSTM-RNN and LSTM-Pep depict peptide sequences as sentences composed of residue tokens, allowing the prediction of residue arrangement via recurrent neural networks. AMPGAN and RLGen involve training a generator and a discriminator using known peptide data, which compete to produce new peptides, followed by the property evaluation tools to filter out inactive sequences. WAE-PSO and AMPGen generate new peptide sequences by sampling from the latent space learned through an encoder-decoder architecture, employing an attribute classifier to filter peptides with desired properties. Furthermore, we utilized PLM-based methods such as ProtGPT2 and AMPTrans as baselines, which are pre-trained on large-scale protein data and subsequently fine-tuned with peptide data for peptide generation. We then extended EvoDiff using pathogen-specific peptide data to enable the generation of peptide sequences with the desired function. Additionally, ProGen employs a conditional transformer as the generative model and uses peptide tags as conditions. This model is pre-trained on protein data and fine-tuned with peptide data labeled with various pathogen information to generate peptides that meet the specified pathogen requirement. ProteoGAN and AMPGAN-v2 use conditional GAN models, controlling generation with conditioning variables for the generator component. Notably, AMPGAN-v2 incorporates domain knowledge of antimicrobial peptides for encoding categorical labels. AR-VAE and HydrAMP employ conditional variational autoencoders as their generative models. Furthermore, AR-VAE uses pathogen labels and HydrAMP utilizes a trainable antimicrobial classifier as conditions for peptide generation. Cut&CLIP adapts the contrastive language–image pre-training (CLIP) framework by leveraging known protein sequences as conditional inputs for peptide generation. In this study, we extend its application by substituting the protein inputs with specific pathogens, enabling the generation of peptides tailored to individual pathogens. PrefixProt employs the pre-trained ProtGPT2 as the generative model and uses a prefix-tuning strategy with pathogen tags to control peptide generation.

### 1.3 Details of the evaluation metrics

We required each of the baseline methods and our KPPepGen to generate 2,000 peptide sequences tailored to the given pathogens. And the evaluation of generated peptides was conducted using the following metrics. For peptide sequences, the similarity score serves as a quantitative measure to assess the degree of resemblance between generated and training sequences for specific pathogens. The low similarity score indicates a high level of novelty in generated sequences. This score was calculated using the Needleman-Wunsch algorithm from the Biopython package[25], with BLOSUM62 as the alignment scoring matrix. The instability score was used to evaluate the degree of peptide instability, relying on the composition of amino acids within the sequence. The transmembrane tendency (TM_tend) score[26] indicates the amino acid transmembrane propensity scale, thereby offering insights into the bacteriostatic capacity of peptides to a certain extent. Subsequently, we evaluated four physicochemical properties of generated peptides, including charge, isoelectric point, hydrophobic, and aromaticity. The instability and TM_tend scores, along with physicochemical properties, were assessed using the modlAMP[27] tool. Furthermore, we employed the STREME[28] tool with parameters (e.g., minimum width of 5, maximum width of 15, and p-value threshold of 0.05) to identify sequence motifs within the training and generated peptides, and the universalmotif tool[29] to evaluate the

similarity between pairwise motifs from these two peptide sets.

For peptide structures, we employed the trRosetta tool[30] to predict their 3D structures from amino acid sequences. The DSSP program[31] is used to extract the secondary structure of peptides and analyze the statistical distribution of structures with helix, sheet, and turn types. Notably, antimicrobial peptides exert their inhibitory effects by binding to specific target proteins of pathogens, thereby disrupting their function[32,33]. Previous studies have demonstrated that screening peptides based on their binding affinity to pathogen target proteins is a viable strategy for AMP discovery[34]. For the docking analysis, the pathogen targets are defined as receptors and remain rigid throughout the docking process. We utilized the ZDOCK tool[35] to assess the binding scores of generated peptides to their respective pathogen targets. Additionally, the AutoDock Vina tool[36] is used to evaluate the binding free energy with general receptors, such as LPS and Lipid II, while the AutoDock CrankPep tool[37] is employed to score interactions with specific targets. Subsequently, we used the modlAMP Python package to extract the helical wheel status of generated peptides and visualized their structures using the open-source PyMOL tool. Antimicrobial peptides are reported to exhibit specificity towards distinct microbial targets by engaging with cell surface components or intracellular proteins of specific pathogens[38,32]. Specifically, the general targets were selected based on structural data from the Protein Data Bank (PDB) for different categories: Gram-positive pathogens (PDB ID: 6YFY), Gram-negative pathogens (PDB ID: 1QFG), and Fungus pathogens (PDB ID: 1ZHS). Notably, only 22 out of 56 pathogens have their target structural information resolved and available in the Protein Data Bank (PDB).

For the pathogen specificity, it is reported that penicillin-binding proteins (PBPs) could be identified as therapeutic targets for peptide-based antimicrobial treatments[39,40]. PBPs exhibit structural variations across different bacterial species, which subsequently influence pathogen-specific inhibition[41]. In this study, we collected 22 pathogens with available specific targets to assess the pathogen specificity of the generated peptides, including *E. coli* (3MZE), *S. aureus* (5M18), *A. baumannii* (3UDI), *E. faecalis* (3E6E), *P. aeruginosa* (3OC2), *K. pneumoniae* (8GPW), *L. monocytogenes* (3ZG8), *B. cereus* (7BN9), *S. pneumoniae* (5OJ1), *M. tuberculosis* (6KGH), *B. subtilis* (7BN9), *S. epidermidis* (8C5B), *E. faecium* (6BSQ), *L. innocua* (3ZG7), *B. licheniformis* (1NRF), *S. typhimurium* (4Q6L), *E. cloacae* (5XHR), *S. enterica* (4Q6V), *C. albicans* (7STO), *S. cerevisiae* (8K3Q), *A. fumigatus* (2XVN), and *A. niger* (6IGY). For example, we selected 2,000 generated peptides guided by the *E. coli* prompt and randomly sampled additional 2,000 peptides from the other 21 pathogens. The pathogen-specific target was subjected to molecular docking with peptides generated using both the self-pathogen prompt (specific interactions) and non-specific prompts from other pathogens (non-specific interactions). The docking scores for various pathogens were evaluated following the same procedure.

### 1.4 Details of the implementation

KPPepGen was implemented in Python 3.9.12 and PyTorch 2.1.2, along with PyTorch-Lightning 1.9.2. We utilized the PubMedBERT model with pre-trained parameters from the "microsoft/BiomedNLP-PubMedBERT-base-uncased-abstract-fulltext" configuration. For the knowledge-aware pre-training process, we employed the PubMedBERT model with the frozen parameters and the trainable sequence encoder, along with the corresponding projection layers for each two encoders with peptide-GO triple data for 500 epochs, utilizing a learning rate of 1e-4. Subsequently, we reloaded the aforementioned sequence encoder, adjusted its learning rate to 5e-5, and trained this pre-training model with peptide-pathogen triple data for 3000 epochs at a learning rate of 1e-4. Notably, the sequence encoder for peptide sequences comprises 8 transformer blocks, each with a hidden state size of 512 and 8 heads for multi-head attention. The projectors, followed by the PubMedBERT model and sequence encoder, are based on a multi-layer perceptron (MLP) architecture with five layers.

After the knowledge-aware pre-training process, we extracted the learned pathogen embeddings from the pathogen knowledge graph as the pathogen prompts and converted these prompts to 64 dimensions with an MLP layer. For the peptide diffusion model, we initialized the input residue representation using the residue types (i.e., one-hot features with 20 classes), the genetic information (i.e., features of the BLOSUM62 matrix), and the physicochemical features from AAindex[42]. Then, we implemented the denoising framework with the hybrid module of a pathogen adapter, a transformer-based sequence encoder pre-trained in the GO/pathogen knowledge, and a noise predictor. The former two components integrate pathogen prompts into the model, guiding the contextual embeddings of residues derived from the peptide sequences. Meanwhile, the latter component maps these embeddings to the noise of residue types, thereby enabling the generation of novel peptides corresponding to the pathogen prompts.

Furthermore, we employed a linear schedule for variances $\beta_t$ with the lowest variance $\beta_1$ =1e-4 and highest variance $\beta_T$ =2e-2 for the diffusion model. The number of diffusion timesteps is set to 1,000. Additionally, we reloaded the parameters of the transformer encoder and fine-tuned it with a learning rate of 5e-5. We utilized the batch size of 1,024 and the Adam optimizer with a learning rate of 1e-4 for other model components to optimize this generative model. The maximum number of training epochs is set as 5,000. The hyperparameter for guidance scale $\omega$ is set to 2 to control the strength of the conditional and unconditional prediction for the controllable peptide generation.

Utilizing the well-trained KPPepGen model, we generated new peptide sequences conditioned on given pathogen prompts.

First, we sampled residue types from the marginal distribution of the corresponding pathogen prompts to construct the initial noise sequences. We performed the independent samplings for peptides with various lengths, ultimately collecting a total of 2,000 noise sequences. Then, each noise sequence, along with its corresponding pathogen prompt, was used as input for the backward diffusion process. The model iteratively predicted the noise at diffusion timestep $t$ and progressively removed the noise of residue types until $t = 0$, thereby generating a new peptide sequence. The peptide diffusion process was configured with a total of 1,000 denoising steps, with a time step interval set to 5.

# 2 Methods

## 2.1 Details of the knowledge-aware pre-training

Gene Ontology is a comprehensive knowledge graph that captures factual biological facts through its hierarchical structures of parent-child GO terms. These structures allow the GO knowledge to represent the intricate features and biological relationships[43]. By associating the peptides with their corresponding GO terms, it can enrich the biological information of peptides with the additional GO knowledge, and enhance the relationships among these peptides. Also, we extended this concept to the pathogen domain by extracting a hierarchical structure of pathogen classes to depict the biological relationships among different pathogens. For example, the parent term 'Pathogen' includes three subclasses: Gram-positive, Gram-negative, and Fungus. Within the Gram-positive category, *S. aureus* and *E. faecalis* could be denoted as child terms. Each pathogen term is associated with the corresponding peptide terms, which serve as peptide instances for the pathogen class. Thus, we defined the pathogen knowledge graph as comprising both pathogen terms and peptide terms to encapsulate their biological relationships.

This knowledge-aware pre-training was designed to ensure that node embeddings encapsulate meaningful biological knowledge by optimizing the relationships within the data. Specifically, it minimized the concordance of the positive and negative triples, effectively distinguishing between relevant and irrelevant associations for peptides. Our study incorporated two distinct types of knowledge graphs: one based on GO and the other on pathogen-related data. The pre-training process was executed in a staged manner. Initially, we focused on pre-training using a comprehensive set of triples derived from large-scale GO annotations, which provide a broad foundation of biological context and enhance the semantic space of peptides. Subsequently, the model underwent a second stage of pre-training on pathogen triples, allowing it to adapt the specialized knowledge relevant to pathogens and distinguish the pathogen-specific biases of peptides.

The GO/pathogen knowledge graphs were constructed from a large number of triples, each triple representing a biological relationship. We defined a triple as $(h, r, t)$, where $h$ and $t$ denote the head and tail entities, respectively, and $r$ represents the relationship between them within the knowledge graph. For the GO knowledge graph, two distinct types of entities were incorporated: $e_{GO}$, representing GO terms, and $e_{peptide}$, representing peptides. The relationships among the $e_{GO}$ entities were categorized into five types: "regulates", "positively_regulates", "negatively_regulates", "is_a", and "part_of." The relationship between $e_{GO}$ and $e_{peptide}$ was represented by a single relation type. All the relation types were encoded as one-hot features for the knowledge graph to maintain uniformity in their representation. Similarly, we also utilized the aforementioned procedure for the pathogen knowledge graph.

To effectively integrate the generic and pathogen-specific knowledge, we employ a two-stage pretraining strategy. In the first stage, the model is pretrained using the generic peptide-GO knowledge, utilizing two NVIDIA RTX 4090 GPUs. In the second stage, the model is fine-tuned using pathogen-specific knowledge. The relevant knowledge graph files and pretrained model checkpoints will be made publicly accessible at our Zenodo repository. Furthermore, the knowledge graph and pretrained model are designed to support future knowledge updates. Specifically, to incorporate novel peptide-pathogen relationships, pathogen names must be standardized following the nomenclature provided by the gcPathogen database[8]. New relationships can then be appended as triples in the designated input file. For pathogens not included among the 56 species in this study, their biological descriptions should be retrieved from Wikipedia and added to the corresponding metadata file. The pretrained model can subsequently be fine-tuned with a small learning rate (5e-5) to update its parameters accordingly.

## 2.2 Details of the virtual screening procedure

Unlike existing peptide classifiers, which primarily assess whether peptides exhibit antimicrobial activity, this section introduces the pathogen classifiers to evaluate the presence of pathogen-specific activity in peptides. Specifically, peptides associated with a particular pathogen type were extracted as pathogen-specific positive samples, while those not associated with this pathogen type were designated as negative samples from the AMP dataset (Section 1.1). These positive and negative peptides for the given pathogen were then randomly partitioned into training, validation, and test sets in an 8:1:1 ratio. For the pathogen classifier, we utilized the pre-trained sequence encoder derived from the pre-training process for the peptide representation (Section 2.1), coupled with a 5-layer MLP to serve as the pathogen predictor.

Additionally, we applied an approach combining the pathogen classifiers and binding affinity ranking to enhance the accuracy of candidate virtual screening in KPPepGen generation. For instance, separate pathogen classifiers were developed for

*E. coli* and *S. aureus*, with the output values serving as the confidence level of antimicrobial activity. Then, peptides for a given pathogen type were ranked based on their binding affinity to pathogen targets, with those exceeding the average affinity being selected for further analysis. For peptides specific to *S. aureus*, we selected those with a confidence level exceeding 0.95 in the *S. aureus* classifier. A similar procedure was followed to identify peptides specific to *E. coli*.

Subsequently, we performed molecular dynamics (MD) simulations using the GROMACS software to evaluate the interactions between peptides and the specific target of the given pathogen. The structure of the peptide-target complex was extracted from the above docking analysis with the best docking score. Then the complex was parameterized using the CHARMM36 all-atom force field and solvated within a dodecahedron box using the SPC water model. The MD simulation workflow comprised four stages: energy minimization, heating, equilibration, and production simulation. Initially, the heavy atoms of the complex system were constrained, and 50,000 steps of energy minimization were performed with a step size of 0.01. The system was then gradually heated to 300 K over 100 ps. Subsequently, equilibration was conducted for an additional 100 ps to stabilize the system. The production MD simulations were then executed for 50 ns with a time step of 2 fs, and trajectory data were recorded at 100 ps intervals. The root-mean-square-deviation (RMSD) of the peptide backbone was calculated using the grmsd package, and the corresponding plots were generated with the xmgrace tool. To evaluate binding affinities, the binding free energy ($\Delta G_{binding}$) of the peptide-target complex was calculated using the g_mmpbsa tool[44], based on the Molecular Mechanics Poisson-Boltzmann Surface Area (MM-PBSA) method. And the conformational stability was evaluated via root-mean-square-fluctuation (RMSF) analyses from simulation trajectories.

### 2.3 Details of the wet-lab evaluation

Peptides were synthesized using the Fmoc-based solid-phase peptide synthesis (SPPS) method, with a purity greater than 98%, by Nanjing GenScript Biotechnology Co., Ltd. The molecular masses of the peptides were further confirmed by electrospray ionization mass spectrometry (ESI-MS), while their purity was assessed using reversed-phase high-performance liquid chromatography (RP-HPLC).

Pathogens used for minimum inhibitory concentration (MIC) measurements included *E. coli* ATCC 25922 and *S. aureus* ATCC 25923. Both pathogens were streaked on Luria–Bertani (LB) agar and incubated at 37°C overnight. Individual colonies were then selected from the agar plates and transferred to Mueller–Hinton broth (MHB) (Hopebio, China) for further cultivation. The cultures were shaken at 160 rpm at 37°C overnight. Once the turbidity of the bacterial suspensions reached between 0.4 and 0.6 McFarland (MCF), the suspensions were diluted 20-fold with MHB. For the preparation of antimicrobial peptide solutions, chemically synthesized peptides were dissolved in either ultrapure water (Biosharp, China) or dimethyl sulfoxide (DMSO) (Biosharp, China) to achieve a stock concentration of 2,560 mg/mL. This stock solution was then further diluted with MHB to obtain the concentration gradient of 2 to 512 $\mu$g/mL for testing. Subsequently, 200 $\mu$L of each peptide dilution was pipetted into 96-well plates, followed by the addition of 10 $\mu$L of the diluted bacterial suspension to each well. The plates were incubated at 37°C for 14-20 hours, and MIC values were determined as the lowest peptide concentration at which no bacterial growth was observed. Antimicrobial activity assays were conducted in 96-well plates with ten experimental groups as follows: 1) blank control group, containing 200 $\mu$L of MHB without bacterial suspension; 2) bacterial control group, with 200 $\mu$L of MHB and 10 $\mu$L of bacterial suspension without peptide; 3) peptide treatment groups, consisting of 200 $\mu$L of MHB containing varying peptide concentration gradient of 2 to 512 $\mu$g/mL along with 10 $\mu$L of bacterial suspension. All experiments were conducted in triplicate. The positive (proper growth of bacteria) and negative (sterility) controls were included.

The Cell Counting Kit-8 (CCK-8) assay was employed to evaluate the cytotoxicity of peptides against human lung epithelial Beas-2B cells. The cells were seeded at a density of $5 \times 10^3$ cells per 100 $\mu$L in 96-well plates and cultured overnight at 37°C with 5% $CO_2$. Subsequently, the cells were treated with 100 $\mu$L of F-12K medium (Procell, China) containing the peptide concentration gradient of 4 to 512 $\mu$g/mL and incubated for 24 hours. After incubation, 10 $\mu$L of CCK-8 solution (Abbkine, China) was added to each well, and the cells were incubated for an additional 4 hours at 37°C. Optical density (OD) at 450 nm was measured using an automated microplate reader (BioTek, USA). Each treatment was performed in six duplicate wells. Cell viability was calculated as the percentage relative to the control, using the formula: Cell viability (%) = [(OD[peptide] - OD[blank]) / (OD[control] - OD[blank])] × 100%.

## 3 Results

### 3.1 More landscape for peptide datasets

For the diversity of GO terms, the biological process category encompasses 3,430 terms (accounting for 47% of the total terms) and 245,683 peptides (representing 22% of the total peptides); the cellular component category includes 819 terms (11%) and 469,675 peptides (43%); the molecular function category covers 3,017 terms (42%) and 389,436 peptides (35%). In the context of pathogen diversity (Table 1), the Gram-positive group comprises 24 distinct pathogens (accounting for 43% of the total pathogen numbers), and 31,726 peptide terms (representing 49% of the total peptide numbers). The Gram-negative group is characterized by 17 pathogens (constituting 30% of the pathogen diversity), and 28,206 peptide terms (making up 44% of the

peptide count). Additionally, the fungus group includes 15 pathogens (27% of the total pathogens), and 4,555 peptide terms (7% to the overall peptide count).

**Table 1.** Statistical profile of peptides across different pathogen types.

| Group | Pathogen types | Number |
|---|---|---|
| Gram-positive | *S. aureus*# (10,341); *B. acillus* (4,639); *B. subtilis*# (3,613); *S. epidermidis*# (2,576); *E. faecium*# (2,194);<br>*E. faecalis*# (2,177); *L. monocytogenes*# (986); *B. cereus*# (941); *B. megaterium* (563); *S. pyogenes* (479);<br>*S. mutans* (418); *M. luteus* (379); *L. lactis* (395); *S. pneumoniae*# (351); *L. innocua*# (260);<br>*B. cinerea* (253); *S. agalactiae* (252); *M. tuberculosis*# (205); *S. uberis* (161); *C. michiganensis* (160);<br>*S. xylosus* (136); *B. anthracis* (134); *B. thuringiensis* (109); *B. licheniformis*# (104); | 24 |
| Gram-negative | *E. coli*# (11,268); *P. aeruginosa*# (7,378); *K. pneumoniae*# (2,671); *A .baumannii*# (1,795);<br>*S. typhimurium*# (1,671); *E. cloacae*# (681); *P. syringae* (369); *S. marcescens* (366); *P. mirabilis* (353);<br>*S. maltophila* (246); *P. vulgaris* (236); *S. enterica*# (218); *V. parahaemolyticus* (219);<br>*V. alginolyticus* (211); *V. parahamelytics* (210); *P. multocida* (159); *X. vesicatoria* (155); | 17 |
| Fungus | *C. albicans*# (1,010); *S. cerevisiae*# (592); *C. parapsilosis* (445); *F. oxysporum* (427); *C. tropicalis* (391);<br>*A. hydrophila* (247); *A. fumigatus*# (236); *C. krusei* (223); *A. niger*# (202); *F. culmorum* (153);<br>*C. glutamicum* (151); *F. solani* (141); *C. sakazakii* (116); *N. crassa* (112); *F. graminearum* (109); | 15 |

Values in parentheses are the corresponding AMP numbers of this pathogen. '#' denotes that the pathogen has an available target structure.

### 3.2 More performance for generation with pathogen prompts

It is clear that antimicrobial peptides and common peptides exhibit marked differences in their physicochemical properties, which in turn shape the distribution of amino acid profiles within the peptide data space[45]. As shown in Figure 1a, our visualizations reveal distinct patterns in the distribution of these amino acid compositions across two peptide groups. Specifically, notable differences become apparent when comparing antimicrobial peptides (AMP) and non-antimicrobial (non-AMP), particularly in the distribution of charged amino acids, such as the prevalence of positive charges represented by arginine (R) and lysine (K), and negative charges by aspartic (D) and glutamic (E).

Herein, we conducted a heatmap analysis on representations of pathogen textual descriptions from the PubMedBert. As shown in Figure 1b, these representations are unable to demonstrate distinct clustering within three pathogen categories and instead exhibit a generalized high correlation across various pathogens. These results suggest a lack of variability among pathogen representations in the semantic space, while the high similarity among pathogen textual descriptions potentially fails to provide effective guidance.

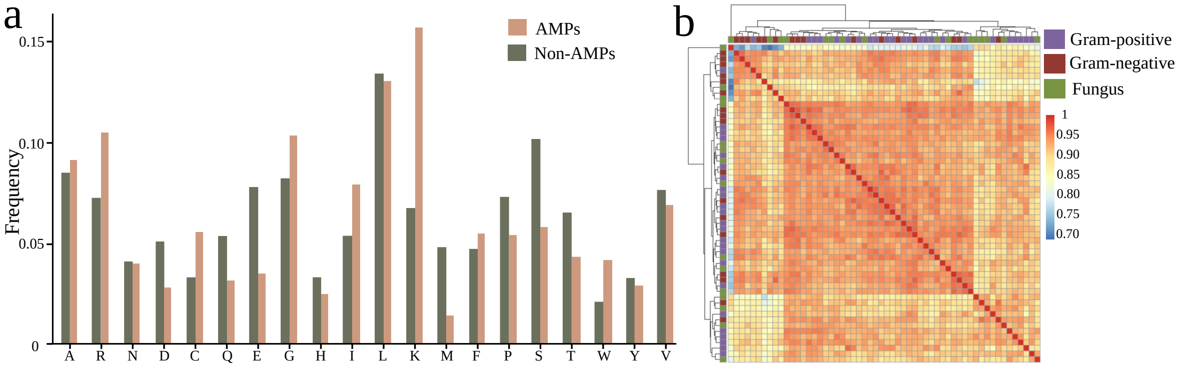


**Figure 1. Additional landscape visualization of peptides. a**, The frequency distribution of amino acids, comparing their occurrence in two peptide groups. **b**, The heatmap depicts the hierarchical clustering relationships among the representations of pathogen textual descriptions across all 56 pathogens in three pathogen categories.

### 3.3 Peptide generation under the zero-shot scenario

In this study, we perform knowledge graph pretraining on both generic knowledge and pathogen-specific knowledge, thereby enriching the peptide sequence space, particularly for pathogens with limited available samples. This knowledge pretraining process also enables the extraction of pathogen prompts to guide downstream peptide generation tasks. Notably, many emerging

or less-studied pathogens lack experimentally validated peptide data. However, relevant biological information, such as GO annotations and functional descriptions of pathogens, is available in existing biomedical knowledge databases. To simulate this challenge, we construct zero-shot learning environments in which the model is not exposed to any peptide data for a given pathogen during training. Instead, the generation model relies solely on a knowledge graph and prompt guidance to generate pathogen-specific peptides.

Specifically, we randomly selected five pathogens, including S. aureus, E. faecalis, L. monocytogenes, S. pneumoniae, and M. tuberculosis. For these selected pathogens, their associated knowledge graph structure was retained for pathogen-specific prompt construction, but their corresponding peptide data were excluded from training in the diffusion-based generative model. As shown in Table 4, we present the peptide generation results for five selected pathogens and evaluate them in comparison with various baseline models. For the KPPepGen with zero-shot setting, there is a notable decline in performance across both sequence- and structure-level metrics compared to the fully trained KPPepGen model. When compared to ProGen, which employs a conditional generation strategy, the zero-shot model exhibits some performance limitations. Nevertheless, in comparison to generative models lacking conditional mechanisms (i.e., EvoDiff, RLGen, and AMPGAN-v2), KPPepGen demonstrates greater performance under the zero-shot scenario. These findings indicate that KPPepGen is capable of generating biologically plausible peptide sequences for previously unseen pathogens by leveraging knowledge graph pretraining and pathogen prompts, thereby highlighting the generalization ability of our model.

**Table 2.** Performance of zero-shot KPPepGen compared to baselines.

| Model | Similarity | Instability | TM_tend | Physicochemical property | | | | General target | Specific target |
|---|---|---|---|---|---|---|---|---|---|
| | | | | Charge | Isoelectric | Hydrophobic | Aromaticity | | |
| EvoDiff | 46.76 | 49.02 | 0.562 | ** | * | *** | ** | 951 | 1074 |
| RLGen | 46.93 | 50.26 | 0.761 | *** | *** | ** | * | 811 | 975 |
| AMPGAN-v2 | 43.25 | 46.21 | 0.558 | *** | ** | * | *** | 920 | 1087 |
| ProGen | 40.81 | 43.77 | 0.531 | * | ns | ns | * | 1042 | 1157 |
| KPPepGen | 34.72 | 40.81 | 0.42 | ns | ns | ns | ns | 1140 | 1289 |
| KPPepGen# | 41.15 | 44.22 | 0.543 | * | * | * | ** | 975 | 1114 |

KPPepGenn# denotes the KPPepGen model evaluated under the zero-shot scenario. ns, no statistical significance; * p<5e-2; ** p<1e-2; *** p<1e-3.

### 3.4 More pattern exploration for generated peptides

It is reported that the arrangement of amino acids in peptides is associated with critical sequence and structure patterns, reflecting the selection pressures exerted by nature[46,47]. The tendency of amino acids to remain unchanged at specific positions often represents key evolutionary information that has been preserved over extended periods[48]. Furthermore, we analyzed the sequence patterns of generated peptides for various pathogens by examining their motifs. As illustrated in Figure 2a, conserved patterns are observed in the amino acid positions within the sites of generated peptides. Specifically, these motifs highlight the regions of significant amino acid conservation, such as the positively charged residues lysine (K) and arginine (R). These conserved regions also showcase the presence of hydrophobic and polar amino acids, including phenylalanine (F), glycine (G), and serine (S). Indeed, these positive residues engage in electrostatic interactions with the negatively charged surfaces of bacterial pathogens, along with hydrophobic residues to facilitate the binding of peptides to bacterial membranes, while polar residues contribute to the overall stability[49,50].

Notably, despite retaining evolutionarily conserved residues critical for antimicrobial efficacy, our generated peptides exhibit substantial sequence diversity, particularly when targeted to specific pathogens. This highlights a finely tuned equilibrium in amino acid composition that is essential for maintaining antimicrobial efficacy. For instance, the localized fragment 'RQRRR' (highlighted in red box) in the corresponding motif is enriched in training peptides of *P. aeruginosa*, with 10 occurrences observed in pathogen-specific sequences compared to 18 occurrences across all peptide data, while our generated peptides could capture this pattern when guided by the corresponding pathogen prompt. Similarly, under the pathogen guidance of *E. coli*, our model identified the key fragment 'KRFIK', further validating its ability to capture pathogen-specific sequence features. The generated peptides expand the original sequence space through the strategic integration of distinct high-frequency fragments, yielding novel combinatorial fragments such as 'ISH' with 'RRYK' and 'GRRK' with 'IHPK'. Consequently, these findings demonstrate that our KPPepGen could effectively identify the key biological information embedded in peptides from the training data of specific pathogens, thereby preserving antimicrobial efficacy in various pathogens.

The interaction of AMPs with bacterial cell walls or membrane components plays a crucial role in determining their efficacy against specific pathogens[51]. It is crucial to recognize these variations in the target proteins across different pathogens, as these variations can significantly influence the efficacy of AMPs. For instance, the specific penicillin-binding proteins across different

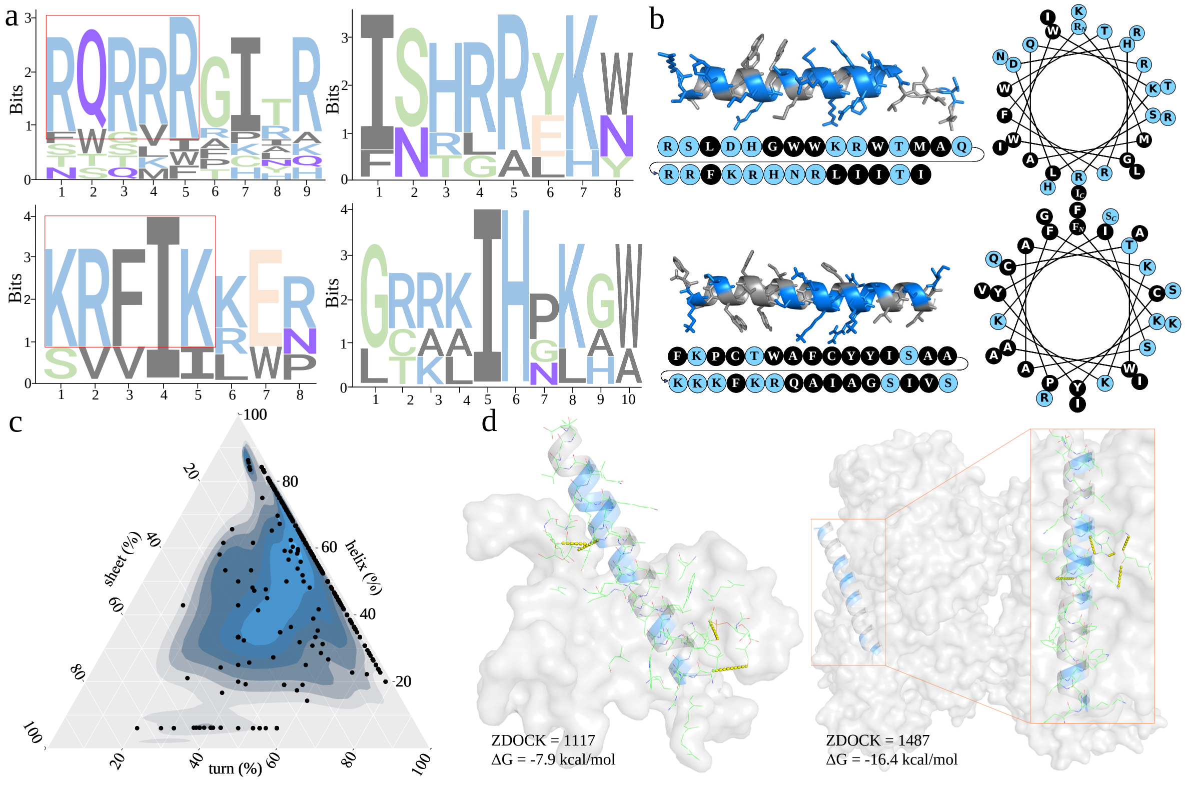


**Figure 2. Additional sequence and structure patterns for our generated peptides. a**, Sequence motifs of generated peptides. **b**, Structural organization of amino acid side chains in generated peptides. The helical wheel plots illustrate the spatial arrangement of amino acids. Hydrophobic and polar residues are highlighted in black and blue. **c**, Ternary diagram illustrating the distribution of secondary structures. The scatter points indicate generated peptides. The density gradient area displays the distribution of training peptides. **d**, Docking posture visualization for generated peptides with general (left, PDB ID: 6YFY) and specific (right, PDB ID: 5M18) targets for *S. aureus*.

pathogens may exhibit varying levels of sensitivity to a given antimicrobial peptide[52]. These mechanisms underscore the ability of AMPs to exhibit selective targeting within pathogens, contributing to their potential therapeutic applications. Herein, we randomly selected the generated peptides with varying lengths from KPPepGen and visualized their conformations in the context of peptide generation for *S. aureus* as an example. As shown in Figure 2b, these peptides predominantly adopt helical structures. Moreover, the helical wheel plots reveal alternating clusters of hydrophobic and polar amino acids, as demonstrated by the spatial arrangement of polar residues (highlighted in blue) maintaining consistent directional orientation within helical structures. This structural characteristic imparts conformational flexibility, allowing peptides to dynamically interface with the biofilm and adapt to a wide variety of structural features across various pathogen targets[53,54]. Subsequently, to evaluate the structural diversity of our generated peptides, we extracted the secondary structure features for peptides of these pathogens, including scores for helix, sheet, and turn formations. We displayed the formation-specific structural compositions of our generated peptides and training peptides for these two pathogens using ternary diagrams. As shown in Figure 2c, the secondary structures of training data are represented as density regions, while those of generated peptides are depicted as scatter plots. Notably, the majority of our generated peptides are concentrated in the helix region and high-density blue region, indicating a similarity in secondary structure distribution between the generated and training peptides. These results demonstrate that our KPPepGen can generate peptides with diverse structures, highlighting the rationality and diversity of the generated peptides across various pathogens.

Furthermore, we selected a generated peptide from the high-density gradient region (Figure 2c), representing the predominant structural pattern of *S. aureus*. Then, we examined the docking postures of this peptide with the general and specific targets and evaluated the binding free energies of the resulting complexes. As illustrated in Figure 2d, our results demonstrate that the peptide exhibits low docking energies, with -7.9 kcal/mol at the general target (PDB ID: 6YFY) and -16.4 kcal/mol at specific (PDB ID: 5M18) targets, alongside the high ZDOCK scores for these targets. These metrics indicate a substantial binding affinity between peptides and their respective pathogen targets. Similar to the docking conformations for *E. coli* in the main manuscript, we extended our docking analysis to peptides targeting *S. aureus*. The side chains of these peptides display

distinct directionality within the target's pocket region, engaging in well-defined interactions with the polar regions on the target surface. These interactions establish multiple hydrogen bonds (i.e., four and five interactions for the general and specific targets), highlighting the strength of their binding affinity. Overall, the above results indicate that our generated peptides for various pathogens suggest their strong binding affinities and interaction patterns with corresponding pathogen targets.

### 3.5 Ablation study

To investigate the necessity and impact of each component in the KPPepGen model, we conducted a series of comparative analyses involving KPPepGen and its several variants: (1) KPPepGen (w/o KG & Prompt) that removes both the knowledge-aware pre-training and the prompt guidance for diffusion generation; (2) KPPepGen (w/o KG) that only removes the knowledge-aware pre-training, and (3) KPPepGen (w/o Prompt) that only removes the prompt guidance for diffusion generation. Our evaluation focused on the generated peptides with property metrics, encompassing sequence similarity, instability index, TM_tend, and four key physicochemical properties, and their binding scores against both general and specific targets.

**Table 3.** Ablation study on the sequence-level results for the property metrics.

| Methods | Similarity | | Instability | | TM_tend | | Charge | | Isoelectric | | Hydrophobic | | Aromaticity | |
|---|---|---|---|---|---|---|---|---|---|---|---|---|---|---|
| | All | Least-10 | All | Least-10 | All | Least-10 | All | Least-10 | All | Least-10 | All | Least-10 | All | Least-10 |
| KPPepGen (w/o KG & Prompt) | 45.33 | 52.14 | 46.26 | 47.33 | 0.561 | 0.608 | ** | *** | * | ** | *** | *** | *** | *** |
| KPPepGen (w/o KG) | 37.16 | 43.08 | 43.75 | 45.66 | 0.517 | 0.616 | * | ** | * | ** | ** | ** | * | ** |
| KPPepGen (w/o Prompt) | 38.25 | 41.74 | 43.18 | 46.23 | 0.529 | 0.593 | ** | ** | ** | * | ** | *** | ** | ** |
| KPPepGen | 34.54 | 37.27 | 40.55 | 42.52 | 0.440 | 0.513 | ns | ns | ns | ns | ns | ns | ns | ns |

**Table 4.** Ablation study on the structure-level results for the docking performance.

| Methods | General target | | Specific target | |
|---|---|---|---|---|
| | 22 pathogens | Least-10 | 22 pathogens | Least-10 |
| KPPepGen (w/o KG & Prompt) | 982 | 964 | 1055 | 1072 |
| KPPepGen (w/o KG) | 1071 | 1009 | 1192 | 1107 |
| KPPepGen (w/o Prompt) | 1058 | 992 | 1176 | 1088 |
| KPPepGen | 1144 | 1097 | 1296 | 1283 |

As demonstrated in Table 2, peptides generated by the full KPPepGen model show lower similarity, instability, and TM_tend scores compared to those produced by the models without knowledge-aware pre-training or prompt guidance. Additionally, the physicochemical properties of peptides generated by different model variants exhibited statistically significant variability. These findings underscore the substantial contributions of the pre-training process and pathogen prompt to enhancing sequence novelty, stability, and effectiveness. Moreover, our results suggest that the impacts of pre-training and prompt are influenced by the dataset size of the studied pathogens. For instance, the pre-training process is especially effective at enhancing sequence novelty when applied to the least-10 peptide datasets. This improvement could be due to the pre-training's ability to integrate and leverage external peptide knowledge, thereby broadening the generalizability of the semantic space even in the context of limited fine-tuning data. Additionally, a similar trend is also observed in the evaluation of peptide structures (Table 3), both for general/specific targets in various pathogens. These results suggest that the pre-training knowledge graph provides a broad, knowledge foundation to enrich the sequence semantic space, while the pathogen prompt process fine-tunes this peptide space for the given requirement, enhancing the performance of peptide generation for specific pathogens.

In summary, our ablation analysis highlights the important roles of knowledge-aware pre-training and prompt guidance for the KPPepGen model, where each component contributes uniquely to the controllable generation of pathogen-specific antimicrobial peptides.

### 3.6 Peptide generation under various length ranges

In drug discovery, shorter peptides are particularly advantageous due to their lower synthesis costs, enhanced cellular permeability, and improved pharmacokinetic properties[55]. As shown in Figure 3a, a length distribution analysis of all antimicrobial peptides (AMPs) revealed that the majority (>79%) consist of 5–25 amino acids. To investigate the competitive binding advantages of peptides relative to small-molecule antibiotics, we selected the penicillin-binding protein (PBP) of E. coli (PDB ID: 3MZE) as a representative example.

Specifically, docking analysis revealed that conventional small-molecule antibiotics, such as cefoxitin and cefuroxime, exhibited binding affinities lower than -10 kcal/mol. In contrast, we randomly selected 50 AMPs for E. coli (ranging in length from 5 to 15 amino acids) from the training set and evaluated their binding affinities, which averaged at -19.1 kcal/mol, underscoring the superior target-binding potential of peptides relative to small molecules. Furthermore, 50 peptides generated by KPPepGen within the same length range exhibited comparable binding energy distributions (-19.6±2.8 kcal/mol). To further investigate the effect of peptide length, we categorized the peptides of E. coli into three groups: (i) Training peptides (5–15 amino acids), (ii) Generated peptides (5-15 amino acids), and (iii) Generated peptides (16–25 amino acids). All selected peptides were docked to the target of E. coli using the ZDOCK tool. As shown in Figure 3b, the results revealed no statistically significant difference in binding scores between the training and generated peptides within the 5-15 length range. Notably, peptides in the 16–25 range from the generated set exhibited a significant improvement in binding scores. These findings suggest that the KPPepGen model is capable of generating peptides with effective target-binding affinities across a range of lengths, thereby demonstrating its applicability in peptide design.

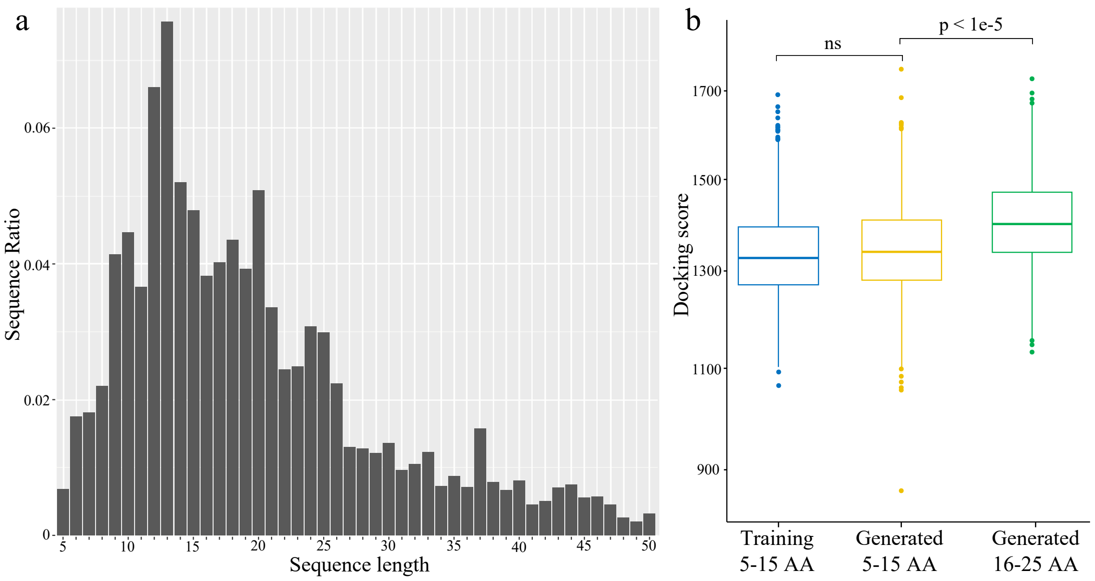


**Figure 3. Peptide sequence length distribution and docking analysis.** **a**, Distribution of peptide sequence lengths in the training set and sequence ratios. **b**, Comparative analysis of docking scores against the E. coli target (PDB ID: 3MZE) for three peptide groups.

## References


1. Wang, G., Li, X. & Wang, Z. APD3: The antimicrobial peptide database as a tool for research and education. *Nucleic Acids Res.* **44**, D1087–D1093, DOI: 10.1093/nar/gkv1278 (2016).
2. Gawde, U. *et al.* CAMPR4: A database of natural and synthetic antimicrobial peptides. *Nucleic Acids Res.* **51**, D377–D383, DOI: 10.1093/nar/gkac933 (2023).
3. Jhong, J.-H. *et al.* dbAMP 2.0: Updated resource for antimicrobial peptides with an enhanced scanning method for genomic and proteomic data. *Nucleic Acids Res.* **50**, D460–D470, DOI: 10.1093/nar/gkab1080 (2022).
4. Shi, G. *et al.* DRAMP 3.0: An enhanced comprehensive data repository of antimicrobial peptides. *Nucleic Acids Res.* **50**, D488–D496, DOI: 10.1093/nar/gkab651 (2022).
5. Singh, S. *et al.* SATPdb: A database of structurally annotated therapeutic peptides. *Nucleic Acids Res.* **44**, D1119–D1126, DOI: 10.1093/nar/gkv1114 (2016).
6. Piotto, S. P., Sessa, L., Concilio, S. & Iannelli, P. YADAMP: Yet another database of antimicrobial peptides. *Int. J. Antimicrob. Agents* **39**, 346–351, DOI: 10.1016/j.ijantimicag.2011.12.003 (2012).
7. Zhao, X., Wu, H., Lu, H., Li, G. & Huang, Q. LAMP: A Database Linking Antimicrobial Peptides. *PLOS ONE* **8**, e66557, DOI: 10.1371/journal.pone.0066557 (2013).
8. Guo, C. *et al.* gcPathogen: A comprehensive genomic resource of human pathogens for public health. *Nucleic Acids Res.* **52**, D714–D723, DOI: 10.1093/nar/gkad875 (2024).

9. Müller, A. T., Hiss, J. A. & Schneider, G. Recurrent Neural Network Model for Constructive Peptide Design. *J. Chem. Inf. Model.* **58**, 472–479, DOI: 10.1021/acs.jcim.7b00414 (2018).
10. Ghorbani, M., Prasad, S., Brooks, B. R. & Klauda, J. B. Deep attention based variational autoencoder for antimicrobial peptide discovery, DOI: 10.1101/2022.07.08.499340 (2022).
11. Madani, A. *et al.* Large language models generate functional protein sequences across diverse families. *Nat. Biotechnol.* **41**, 1099–1106, DOI: 10.1038/s41587-022-01618-2 (2023).
12. Alamdari, S. *et al.* Protein generation with evolutionary diffusion: Sequence is all you need, DOI: 10.1101/2023.09.11.556673 (2023).
13. Mardikoraem, M., Wang, Z., Pascual, N. & Woldring, D. Generative models for protein sequence modeling: Recent advances and future directions. *Briefings Bioinforma.* **24**, bbad358, DOI: 10.1093/bib/bbad358 (2023).
14. Mao, J. *et al.* Application of a deep generative model produces novel and diverse functional peptides against microbial resistance. *Comput. Struct. Biotechnol. J.* **21**, 463–471, DOI: 10.1016/j.csbj.2022.12.029 (2023).
15. Zhang, H. *et al.* Deep Learning-Based Bioactive Therapeutic Peptide Generation and Screening. *J. Chem. Inf. Model.* **63**, 835–845, DOI: 10.1021/acs.jcim.2c01485 (2023).
16. Lin, T.-T. *et al.* Discovering Novel Antimicrobial Peptides in Generative Adversarial Network, DOI: 10.1101/2021.11.22.469634 (2021).
17. Wang, R. *et al.* Diff-AMP: Tailored designed antimicrobial peptide framework with all-in-one generation, identification, prediction and optimization. *Briefings Bioinforma.* **25**, bbae078, DOI: 10.1093/bib/bbae078 (2024).
18. Yang, L. *et al.* Accelerating the discovery of anticancer peptides targeting lung and breast cancers with the Wasserstein autoencoder model and PSO algorithm. *Briefings Bioinforma.* **23**, bbac320, DOI: 10.1093/bib/bbac320 (2022).
19. Kucera, T., Togninalli, M. & Meng-Papaxanthos, L. Conditional generative modeling for de novo protein design with hierarchical functions. *Bioinformatics* **38**, 3454–3461, DOI: 10.1093/bioinformatics/btac353 (2022).
20. Oort, C. M. V., Ferrell, J. B., Remington, J. M., Wshah, S. & Li, J. AMPGAN v2: Machine Learning-Guided Design of Antimicrobial Peptides. *J. Chem. Inf. Model.* DOI: 10.1021/acs.jcim.0c01441 (2021).
21. Szymczak, P. *et al.* Discovering highly potent antimicrobial peptides with deep generative model HydrAMP. *Nat. Commun.* **14**, 1453, DOI: 10.1038/s41467-023-36994-z (2023).
22. Hawkins-Hooker, A. *et al.* Generating functional protein variants with variational autoencoders. *PLOS Comput. Biol.* **17**, e1008736, DOI: 10.1371/journal.pcbi.1008736 (2021).
23. Palepu, K. *et al.* Design of Peptide-Based Protein Degraders via Contrastive Deep Learning, DOI: 10.1101/2022.05.23.493169 (2022).
24. Luo, J., Liu, X., Li, J., Chen, Q. & Chen, J. Controllable Protein Design by Prefix-Tuning Protein Language Models, DOI: 10.1101/2023.12.03.569747 (2024).
25. Cock, P. J. A. *et al.* Biopython: Freely available Python tools for computational molecular biology and bioinformatics. *Bioinformatics* **25**, 1422–1423, DOI: 10.1093/bioinformatics/btp163 (2009).
26. Zhao, G. & London, E. An amino acid “transmembrane tendency” scale that approaches the theoretical limit to accuracy for prediction of transmembrane helices: Relationship to biological hydrophobicity. *Protein Sci.* **15**, 1987–2001, DOI: 10.1110/ps.062286306 (2006).
27. Müller, A. T., Gabernet, G., Hiss, J. A. & Schneider, G. modlAMP: Python for antimicrobial peptides. *Bioinformatics* **33**, 2753–2755, DOI: 10.1093/bioinformatics/btx285 (2017).
28. Bailey, T. L. STREME: Accurate and versatile sequence motif discovery. *Bioinformatics* **37**, 2834–2840, DOI: 10.1093/bioinformatics/btab203 (2021).
29. Tremblay, B. J.-M. Universalmotif: An R package for biological motif analysis. *J. Open Source Softw.* **9**, 7012, DOI: 10.21105/joss.07012 (2024).
30. Yang, J. *et al.* Improved protein structure prediction using predicted interresidue orientations. *Proc. Natl. Acad. Sci.* **117**, 1496–1503, DOI: 10.1073/pnas.1914677117 (2020).
31. Touw, W. G. *et al.* A series of PDB-related databanks for everyday needs. *Nucleic Acids Res.* **43**, D364–D368, DOI: 10.1093/nar/gku1028 (2015).
32. Yao, L. *et al.* dbAMP 3.0: Updated resource of antimicrobial activity and structural annotation of peptides in the post-pandemic era. *Nucleic Acids Res.* **53**, D364–D376, DOI: 10.1093/nar/gkae1019 (2025).
33. Vidal-Limon, A., Aguilar-Toalá, J. E. & Liceaga, A. M. Integration of Molecular Docking Analysis and Molecular Dynamics Simulations for Studying Food Proteins and Bioactive Peptides. *J. Agric. Food Chem.* **70**, 934–943, DOI: 10.1021/acs.jafc.1c06110 (2022).
34. Mustafa, G., Mehmood, R., Mahrosh, H. S., Mehmood, K. & Ahmed, S. Investigation of Plant Antimicrobial Peptides against Selected Pathogenic Bacterial Species Using a Peptide-Protein Docking Approach. *BioMed Res. Int.* **2022**, 1077814, DOI: 10.1155/2022/1077814 (2022).

35. Pierce, B. G., Hourai, Y. & Weng, Z. Accelerating Protein Docking in ZDOCK Using an Advanced 3D Convolution Library. *PLOS ONE* **6**, e24657, DOI: 10.1371/journal.pone.0024657 (2011).
36. Eberhardt, J., Santos-Martins, D., Tillack, A. F. & Forli, S. AutoDock Vina 1.2.0: New Docking Methods, Expanded Force Field, and Python Bindings. *J. Chem. Inf. Model.* **61**, 3891–3898, DOI: 10.1021/acs.jcim.1c00203 (2021).
37. Zhang, Y. & Sanner, M. F. AutoDock CrankPep: Combining folding and docking to predict protein–peptide complexes. *Bioinformatics* **35**, 5121–5127, DOI: 10.1093/bioinformatics/btz459 (2019).
38. Savini, F. *et al.* Binding of an antimicrobial peptide to bacterial cells: Interaction with different species, strains and cellular components. *Biochimica et Biophys. Acta (BBA) - Biomembr.* **1862**, 183291, DOI: 10.1016/j.bbamem.2020.183291 (2020).
39. Zou, Q. & Yang, K.-L. Identification of peptide inhibitors of penicillinase using a phage display library. *Anal. Biochem.* **494**, 4–9, DOI: 10.1016/j.ab.2015.10.009 (2016).
40. Tang, H. W., Phapugrangkul, P., Fauzi, H. M. & Tan, J. S. Lactic Acid Bacteria Bacteriocin, an Antimicrobial Peptide Effective Against Multidrug Resistance: A Comprehensive Review. *Int. J. Pept. Res. Ther.* **28**, 14, DOI: 10.1007/s10989-021-10317-6 (2021).
41. Xu, X. *et al.* In silico screening of protein-binding peptides with an application to developing peptide inhibitors against antibiotic resistance. *PNAS Nexus* **3**, pgae541, DOI: 10.1093/pnasnexus/pgae541 (2024).
42. Kawashima, S. *et al.* AAindex: Amino acid index database, progress report 2008. *Nucleic Acids Res.* **36**, D202–D205, DOI: 10.1093/nar/gkm998 (2008).
43. The Gene Ontology Consortium *et al.* The Gene Ontology knowledgebase in 2023. *Genetics* **224**, iyad031, DOI: 10.1093/genetics/iyad031 (2023).
44. Kumari, R., Kumar, R. & Lynn, A. G_mmpbsa—A GROMACS Tool for High-Throughput MM-PBSA Calculations. *J. Chem. Inf. Model.* **54**, 1951–1962, DOI: 10.1021/ci500020m (2014).
45. Szymczak, P. & Szczurek, E. Artificial intelligence-driven antimicrobial peptide discovery. *Curr. Opin. Struct. Biol.* **83**, 102733, DOI: 10.1016/j.sbi.2023.102733 (2023).
46. Schmitt, P., Rosa, R. D. & Destoumieux-Garzón, D. An intimate link between antimicrobial peptide sequence diversity and binding to essential components of bacterial membranes. *Biochimica et Biophys. Acta (BBA) - Biomembr.* **1858**, 958–970, DOI: 10.1016/j.bbamem.2015.10.011 (2016).
47. Shafee, T. M. A., Lay, F. T., Phan, T. K., Anderson, M. A. & Hulett, M. D. Convergent evolution of defensin sequence, structure and function. *Cell. Mol. Life Sci.* **74**, 663–682, DOI: 10.1007/s00018-016-2344-5 (2017).
48. Celniker, G. *et al.* ConSurf: Using Evolutionary Data to Raise Testable Hypotheses about Protein Function. *Isr. J. Chem.* **53**, 199–206, DOI: 10.1002/ijch.201200096 (2013).
49. Travkova, O. G., Moehwald, H. & Brezesinski, G. The interaction of antimicrobial peptides with membranes. *Adv. Colloid Interface Sci.* **247**, 521–532, DOI: 10.1016/j.cis.2017.06.001 (2017).
50. Torres, M. D. T., Sothiselvam, S., Lu, T. K. & de la Fuente-Nunez, C. Peptide Design Principles for Antimicrobial Applications. *J. Mol. Biol.* **431**, 3547–3567, DOI: 10.1016/j.jmb.2018.12.015 (2019).
51. Gan, B. H., Gaynord, J., Rowe, S. M., Deingruber, T. & Spring, D. R. The multifaceted nature of antimicrobial peptides: Current synthetic chemistry approaches and future directions. *Chem. Soc. Rev.* **50**, 7820–7880, DOI: 10.1039/D0CS00729C (2021).
52. Kumar, N., Sood, D., Tomar, R. & Chandra, R. Antimicrobial Peptide Designing and Optimization Employing Large-Scale Flexibility Analysis of Protein-Peptide Fragments. *ACS Omega* **4**, 21370–21380, DOI: 10.1021/acsomega.9b03035 (2019).
53. Liang, Y., Zhang, X., Yuan, Y., Bao, Y. & Xiong, M. Role and modulation of the secondary structure of antimicrobial peptides to improve selectivity. *Biomater. Sci.* **8**, 6858–6866, DOI: 10.1039/D0BM00801J (2020).
54. Kabelka, I. & Vácha, R. Advances in Molecular Understanding of $\alpha$-Helical Membrane-Active Peptides. *Accounts Chem. Res.* **54**, 2196–2204, DOI: 10.1021/acs.accounts.1c00047 (2021).
55. Apostolopoulos, V. *et al.* A Global Review on Short Peptides: Frontiers and Perspectives. *Molecules* **26**, 430, DOI: 10.3390/molecules26020430 (2021).
